# Effect of dietary restriction and subsequent re-alimentation on the transcriptional profile of hepatic tissue in cattle

**DOI:** 10.1186/s12864-016-2578-5

**Published:** 2016-03-17

**Authors:** Kate Keogh, David A. Kenny, Paul Cormican, Alan K. Kelly, Sinead M. Waters

**Affiliations:** Animal and Bioscience Research Department, Animal and Grassland Research and Innovation Centre, Teagasc, Dunsany, Co. Meath, Ireland; UCD School of Agriculture and Food Science, Belfield, Dublin 4, Ireland

**Keywords:** Dietary restriction, Compensatory growth, Cattle, Liver, RNAseq

## Abstract

**Background:**

Compensatory growth (CG) is an accelerated growth phenomenon observed in animals upon re-alimentation following a period of dietary restriction. It is typically utilised in livestock systems to reduce feed costs during periods of reduced feed availability. The biochemical mechanisms controlling this phenomenon, however, are yet to be elucidated. This study aimed to uncover the molecular mechanisms regulating the hepatic expression of CG in cattle, utilising RNAseq. RNAseq was performed on hepatic tissue of bulls following 125 days of dietary restriction (RES) and again following 55 days of subsequent re-alimentation during which the animals exhibited significant CG. The data were compared with those of control animals offered the same diet on an ad libitum basis throughout (ADLIB). Elucidation of the molecular control of CG may yield critical information on genes and pathways which could be targeted as putative molecular biomarkers for the selection of animals with improved CG potential.

**Results:**

Following a period of differential feeding, body-weight and liver weight were 161 and 4 kg higher, respectively, for ADLIB compared with RES animals. At this time RNAseq analysis of liver tissue revealed 1352 significantly differentially expressed genes (DEG) between the two treatments. DEGs indicated down-regulation of processes including nutrient transport, cell division and proliferation in RES. In addition, protein synthesis genes were up-regulated in RES following a period of restricted feeding. The subsequent 55 days of ad libitum feeding for both groups resulted in the body-weight difference reduced to 84 kg, with no difference in liver weight between treatment groups. At the end of 55 days of unrestricted feeding, 49 genes were differentially expressed between animals undergoing CG and their continuously fed counterparts. In particular, hepatic expression of cell proliferation and growth genes were greater in animals undergoing CG.

**Conclusions:**

Greater expression of cell cycle and cell proliferation genes during CG was associated with a 100 % recovery of liver weight during re-alimentation. Additionally, an apparent up-regulation in capacity for cellular protein synthesis during restricted feeding may contribute to and sustain CG during re-alimentation. DEGs identified are potential candidate genes for the identification of biomarkers for CG, which may be incorporated into future breeding programmes.

**Electronic supplementary material:**

The online version of this article (doi:10.1186/s12864-016-2578-5) contains supplementary material, which is available to authorized users.

## Background

As feed can account for up to 75 % of the variable costs in beef cattle production systems [1, 2], any means by which these costs may be reduced without compromising overall feed efficiency or animal performance would be of benefit to the beef industry worldwide. Compensatory growth (CG) is defined as a physiological process whereby an animal has the potential, following a period of restricted feed intake, to undergo accelerated growth upon re-alimentation [3]. The CG phenomenon is commonly utilised by cattle producers to reduce the overwintering costs of beef cattle [4]. However, despite extensive utilisation by producers, there is little understanding of the biological and molecular mechanisms regulating the exaggerated growth phenotype typically observed.

Although typically attributing to between 1 and 1.3 % of body-weight, the liver is a major metabolic organ, accounting for on average, 24 % of whole body energy use [5, 6]. The energy requirement arises from activities associated with absorption and transportation of nutrients for subsequent use by other tissues and also a large portion of this energy is used for the maintenance of tissue integrity and mass [7]. Alterations in the size of the liver have been shown to be directly proportional to dietary intake [8]. Indeed previous work, including our own data, has shown a reduction in the weight and metabolic activity of this organ during dietary restriction, which facilitates efficient coping with restricted nutrient availability, primarily through a reduction in its basal metabolic rate [9–12]. It is thought that this reduced metabolic rate may continue into the initial stages of re-alimentation and thus facilitate the CG process [13]. During subsequent re-alimentation induced CG, the liver has been shown to be one of the most responsive tissues to re-alimentation, compensating ahead of other organs and tissues in the body [9–12]. Indeed liver tissue of the compensating animals in the current study were found to have achieved 100 % recovery following 55 days of re-alimentation, whereas overall body-weight CG index for animals undergoing CG during the same time was only 48 %. A previous microarray based examination of hepatic gene expression during feed restriction, followed by early phase re-alimentation of cattle, has been reported by Connor et al. [14]. The authors noted alterations in the expression of genes associated with cellular division and mitochondrial function during early CG. Next generation RNAseq technology has distinct advantages over microarray technology, including sensitive unbiased detection of all expressed genes without the requirement to generate an array of probes based on known sequence as well as having a much greater dynamic range [15]. Indeed, previous work from our own group using hepatic tissue comparing both microarray and RNAseq datasets pertaining to the same biological samples identified a greater number of DEGs through utilisation of next generation sequencing technology [16]. Furthermore, in the study of Connor et al. [14] differential expression of genes was evaluated within the first 2 weeks of re-alimentation, which potentially may have been too early to identify genes associated with more sustained and lasting CG as genes identified as differentially expressed by Connor et al. [14] may have reflected latent effects of the previous dietary restriction phase. Therefore the objective of the current study was to examine the differential expression of hepatic genes in cattle following an industry typical period of restricted feeding (125 days) and subsequent CG using RNAseq technology. The liver was chosen as a target tissue of interest, as it is a highly metabolic organ and is clearly physically affected by restricted feeding and subsequent re-alimentation induced CG [9, 11]. Our efforts during re-alimentation were focussed within the first 60 days as this is the period where the greatest increment of overall body CG is typically observed [3].

## Methods

All procedures involving animals were approved by the University College Dublin, Animal Research Ethics Committee and licensed by the Irish Department of Health and Children in accordance with the European Community Directive 86/609/EC.

### Animal model

This study was conducted as part of a larger research programme designed to physiologically characterise the effect of restricted growth and subsequent re-alimentation in Holstein Friesian bulls [9, 10]. Briefly, sixty purebred Holstein Friesian bulls with a mean (SEM) age of 479 (15) days and body-weight 370 (35) kg were blocked according to weight, age, sire and a pre-trial body-weight gain into one of two groups: (i) restricted feed allowance for 125 days (RES; *n* = 30) followed by ad libitum access to feed for a further 55 days or (ii) ad libitum access to feed throughout (ADLIB; *n* = 30). The first 125 days was denoted as Period 1 and the subsequent 55 days, Period 2. Period 1 was designed to reflect an industry typical period of dietary restriction of 125 days, whereas 55 days of re-alimentation in Period 2 was designed to capture the peak of CG expression [3]. All animals were offered a total mixed ration diet consisting of 70 % concentrate and 30 % grass silage on a dry matter basis. All animals received the same diet throughout each period, but with different proportions offered depending on treatment group. Diets were offered individually, with the proportion of feed required based on each animal’s own individual body-weight. Animals were weighed on two days at the start of the study, at the end of Period 1 and again at the end of Period 2. Additionally, throughout the study, animals were weighed every 2 weeks during Period 1 and every week during Period 2. Weighing was at the same time each morning before fresh feed was offered. During Period 1 RES animals were managed to achieve a target mean daily growth rate of 0.6 kg/day, based on dietary energy calculations using NRC [1]. At the end of this period 15 animals from each treatment were slaughtered. All remaining animals were slaughtered at the end of Period 2. At each time point slaughter order was randomised to account for potential confounding effects on treatment outcomes.

### Hepatic tissue collection

All animals were slaughtered in an EU licensed abattoir (Euro Farm Foods, Duleek, Co. Meath). Hepatic tissue was sampled from all animals within 30 min of slaughter. All tissue samples were sampled from the same location in each liver. All surgical instruments used for tissue collection were sterilized and treated with RNA Zap prior to use (Ambion, Applera Ireland, Dublin, Ireland). Samples were washed thoroughly with sterile DPBS and immediately snap frozen in liquid nitrogen before subsequent storage at −80 °C.

### RNA isolation and purification

Total RNA was isolated from liver tissue samples using the Qiagen RNeasy mini kit (Qiagen), according to the manufacturer’s instructions. Approximately 60 mg of frozen tissue was used for RNA extraction. The quantity of the RNA isolated was determined by measuring the absorbance at 260 nm using a Nanodrop spectrophotometer ND-1000 (Nanodrop Technologies, DE, USA). RNA quality was assessed on the Agilent Bioanalyser 2100 using the RNA 6000 Nano Lab Chip kit (Agilent Technologies Ireland Ltd., Dublin, Ireland). RNA quality was also verified by ensuring all RNA samples had an absorbance (A260/280) of between 1.8 and 2. RNA samples with 28S/18S ratios ranging from 1.8 to 2.0 and an RNA integrity number of between 8 and 10 were deemed to be of sufficiently high quality. High quality RNA samples were selected from 10 representative animals from each treatment within each period.

### cDNA library preparation and sequencing

cDNA libraries were prepared from high quality RNA using an Illumina TruSeq RNA sample prep kit following the manufacturer’s instructions (Illumina, San Diego, CA, USA). For each sample, 3 μg of total RNA was used for cDNA preparation. Briefly, mRNA was purified from total RNA and then fragmented. First strand cDNA synthesis was performed using SuperScript II Reverse Transcriptase (Applied Biosystems Ltd.) subsequently synthesising the second strand using components of the Illumina TruSeq RNA samples prep kit. Adaptors were ligated to the cDNA which was then enriched by PCR. Final individual cDNA libraries were validated on the Agilent Bioanalyser 2100 using the DNA 1000 Nano Lab Chip kit, ensuring that library fragment size was ~260 bp and library concentration was >30 ng/μl. After quality control procedures, individual RNAseq libraries were pooled based on their respective sample-specific-6 bp adaptors and sequenced at 100 bp/sequence single-end reads using an Illumina HiSeq 2000 sequencer. Approximately 16 million sequences per sample (Mean ± SD = 15,964,874 ± 1,903,207) were generated.

### RNAseq data analyses

Raw sequence reads were first checked for quality using FASTQC software (version 0.10.0). Input reads were then aligned to the bovine reference genome (UMD3.1) using TopHat (v2.0.9). The software package HTSeq (v0.5.4p5) (http://pypi.python.org/pypi/HTSeq) was employed to calculate the number of sequence reads overlapping all protein coding genes from the ENSEMBL v74 annotation of the bovine genome. The number of read counts mapping to each annotated gene from HTSeq was then collated into a single file and used for subsequent differential gene expression. Only uniquely mapped reads were used for subsequent differential gene expression analysis. The R (v2.14.1) Bioconductor package, EdgeR (v3.4.1), which uses a negative binomial distribution model to account for both biological and technical variation, was applied to identify statistically significant differentially expressed genes (DEGs). Reads were first filtered before subsequent differential gene expression analysis, a gene was deemed to be expressed if the number of reads per gene per animal was ≥4. The analysis was undertaken using moderated tagwise dispersions. DEGs are defined as having a Benjamini and Hochberg false discovery rate of < 0.05 % and a fold change cut-off of 1.25 was used for each time-point.

### Pathway analysis

In RNAseq experiments the differences in transcript length can yield different levels of total reads, even if transcripts are expressed at the same level. GOseq is an application for performing gene ontology analysis on RNAseq data while appropriately incorporating the effect of this transcript length selection bias [17]. Biological pathways that were over-represented (*P* < 0.05) among DEGs were identified using the GOseq software package (v.1.14.0 and Kyoto Encyclopaedia of Genes and Genomes (KEGG)). Pathways were deemed over-represented when there were more DEGs in the pathway than would be expected given the size and gene length distribution [17]. Due to the incomplete functional annotation of the bovine genome, to facilitate GOseq analysis, the online tool BioMart (www.ensembl.org/biomart/martview) was used to convert bovine gene IDs to their human orthologs. The resultant set of DEGs was then applied to test KEGG pathways (http://www.genome.jp/kegg/pathway.html) for over- or under-representation. The significant KEGG pathway maps were examined for significant DEGs. To examine the molecular functions and genetic networks, the RNAseq data were further analysed using Ingenuity Pathway Analysis (v. 8.8, Ingenuity Systems, Mountain View, CA; http://www.ingenuity.com), a web-based software application that enables identification of over-represented biological mechanisms, pathways and functions most relevant to experimental datasets or genes of interest [18–21].

### qRT-PCR validation of RNAseq data

The RNAseq results were validated against gene expression values obtained from the same animals used in the current study on component genes of the somatotropic axis which has been described previously by Keogh et al. [22]. These genes represented genes that were identified as both significantly differentially expressed as well as those not affected by either dietary restriction and subsequent re-alimentation induced CG. Briefly, using the same RNA samples that were analysed in the current RNAseq study, cDNA was synthesised and the expression of genes of the somatotropic axis examined using qRT-PCR following both dietary restriction and subsequent re-alimentation. Expression levels of candidate genes (*SOCS3*, *JAK2*, *STAT5B*, *IGF1*, *IGFBP1*-*6*, *ALS*, *GHR1A*) were normalised against expression values of selected hepatic reference genes (*ACTB*, *CAP1*). Gene expression data were checked for normality using the UNIVARIATE procedure of SAS (SAS Inst. Inc., Cary, NC). Where necessary, data were transformed using the Transreg procedure by raising values to the power of λ. Data were analysed using mixed models methodology (PROC MIXED, SAS). The Tukey critical difference test was performed to determine the existence of statistical differences between treatment mean values. The CORR procedure of SAS was used to determine correlations between RNAseq and qRT-PCR data. Pearson correlation coefficients were estimated for each individual gene across all animals. A *P* < 0.05 was considered to be statistically significant.

## Results

### Animal performance

**Fig. 1:**
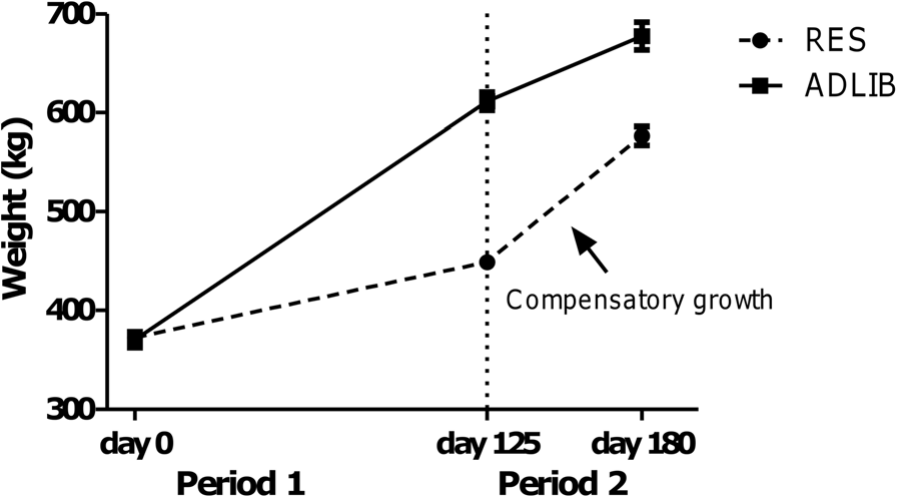
Schematic representation of growth rate and weight gain of the experimental trial showing planned growth paths for ad libitum (ADLIB) and feed restricted-refed (RES) animals

Differences in daily body-weight gain, feed intake and animal performance are outlined in detail by Keogh et al. [9]. Briefly, at the end of 125 days of differential feeding (Period 1), there was a 161 kg body-weight difference between RES and ADLIB groups (442 v 603 kg, respectively; *P* < 0.001). Following 55 days of ad libitum feeding for both groups in Period 2, the body-weight difference was reduced to 84 kg (594 and 678 kg for RES and ADLIB, respectively; *P* < 0.01). Thus during Period 1 body-weight gain was 0.6 kg/day in the RES animals and 1.9 kg/day in the ADLIB animals while during Period 2, animals gained 2.5 and 1.4 kg/day in RES and ADLIB groups, respectively (*P* < 0.001). A schematic growth curve for both RES and ADLIB animals is presented in Fig. 1. Liver weight of animals in RES at the end of Period 1 was only 0.65 of that of their ADLIB contemporaries (RES *v* ADLIB: 4.5 *v* 8.5 kg; *P* < 0.05), while there was no difference in liver weight between treatment groups following 55 days of re-alimentation at the end of Period 2 (RES *v* ADLIB: 8.5 *v* 8.7 kg; *P* > 0.05). Feed intake was less in RES animals compared with ADLIB animals during Period 1 (*P* < 0.001), however during Period 2, no difference in intake between treatment groups was evident (*P* > 0.05). When expressed as a proportion of body-weight, feed intake was greater in animals undergoing re-alimentation and compensatory growth compared to ADLIB animals during the same period (*P* > 0.001). Additionally feed conversion ratio, the ratio between average daily feed intake and average daily gain, which can be used as a measure of feed efficiency was better in RES animals during Period 2 whilst undergoing CG compared with ADLIB animals over both periods (*P* < 0.001). RES animals also displayed reduced fat covering following a 55 day period of re-alimentation induced CG compared with ADLIB animals (fat cover scores: RES *v* ADLIB: 5.1 *v* 7.6 *P* < 0.05).

### mRNA read alignment and differential gene expression

**Fig. 2:**
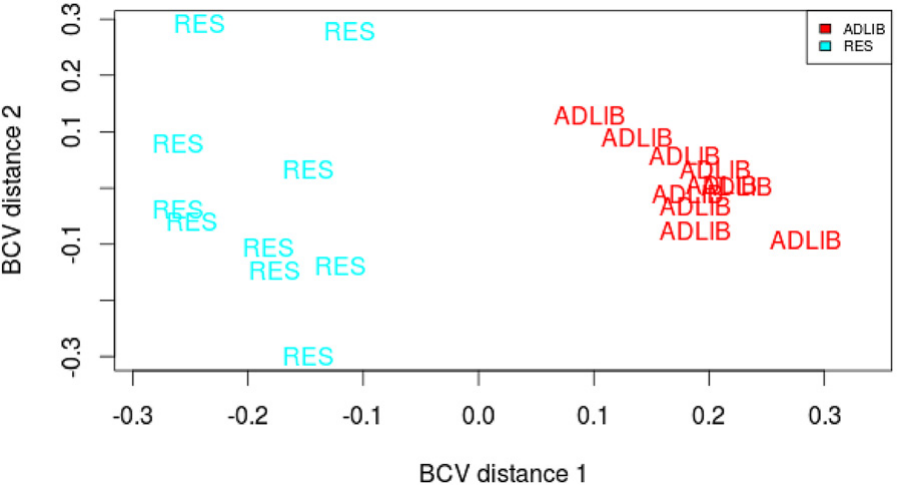
Multi-dimensional scaling plot of hepatic transcript reads following a period of dietary restriction at the end of Period 1. Plot in which distance corresponds to the biological coefficient of variation, with clear separation of RES (*blue*) and ADLIB (*red*) treatment groups in gene transcript abundance reads following a period of restricted feeding at the end of Period 1

**Fig. 3:**
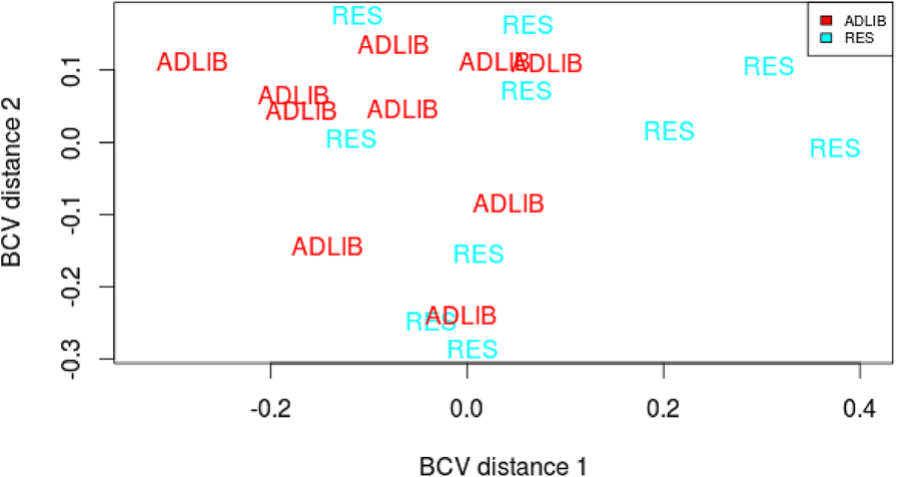
Multi-dimensional scaling plot of hepatic transcript reads following a period of compensatory growth at the end of Period 2. Plot in which distance corresponds to the biological coefficient of variation, with no clear separation between RES (*blue*) and ADLIB (*red*) treatment groups in gene transcript abundance reads following a period of compensatory growth at the end of Period 2

The average (SD) number of raw reads across all samples was 16.6 million (SD = 1.9 million). Approximately 89.5 % of reads aligned to the bovine genome and 77 % of those that aligned were mapped to the gene space. The bovine reference genome (UMD3.1) contains 26,740 gene transcripts. At the end of dietary restriction in Period 1, the number of genes that had mapped reads was 12,150, whereas following 55 days of re-alimentation in Period 2, 12,305 genes had reads mapping to them. A total of 1352 and 49 genes were identified as differentially expressed between RES and ADLIB in periods 1 and 2, respectively. These were manifested as 662 genes with increased and 690 genes with decreased expression in RES relative to ADLIB in Period 1. During CG in Period 2, 26 and 23 genes exhibited increased and decreased expression, respectively, in hepatic tissue of RES compared with ADLIB animals. Figure 2 displays a multi-dimensional scaling plot based on normalised expression values in both RES and ADLIB animals following dietary restriction, with clear separation evident between treatment groups. Following 55 days of subsequent re-alimentation and CG, however, there was little evidence for divergence between the two treatment groups (Fig. 3). Differential gene expression data are consistent with these plots, where a large number of genes were differentially expressed between RES and ADLIB in Period 1, while this was greatly reduced in Period 2. Nine DEG between RES and ADLIB were common across both study periods. These genes included: *COL1A2, DDIT3, DNAJB11, HERPUD1, MANF, PPP1R1B, RPS27, SELK* and *SEMA4B*. However, the direction of the fold change for each of these genes was reversed between the two periods, with the exception of *PPP1R1B* which followed the same pattern for both periods. RNAseq data from the current study are available on NCBI’s Gene Expression Omnibus [23] through GEO Series accession number GSE64285.

### Pathway analysis

**Fig. 4:**
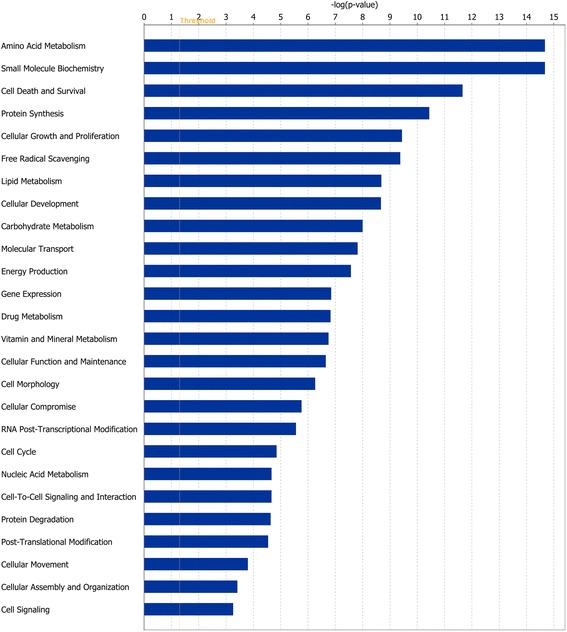
Classification of differentially expressed genes according to molecular and cellular function, most significantly affected by restricted feeding in Period 1. The bars indicate the likelihood [−log (*P* value)] that the specific molecular and cellular function was affected by restricted feeding compared with others represented in the list of differentially expressed genes

**Fig. 5:**
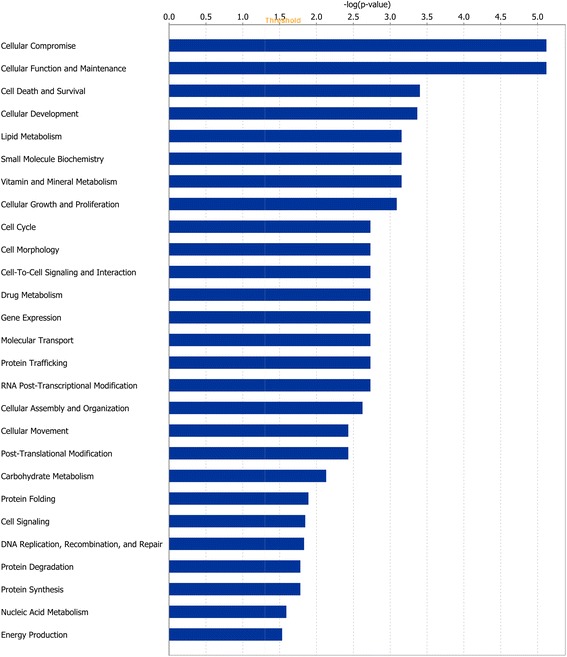
Classification of differentially expressed genes according to molecular and cellular function, most significantly affected by re-alimentation in Period 2. The bars indicate the likelihood [−log (*P* value)] that the specific molecular and cellular function was affected by re-alimentation compared with others represented in the list of differentially expressed genes

**Fig. 6:**
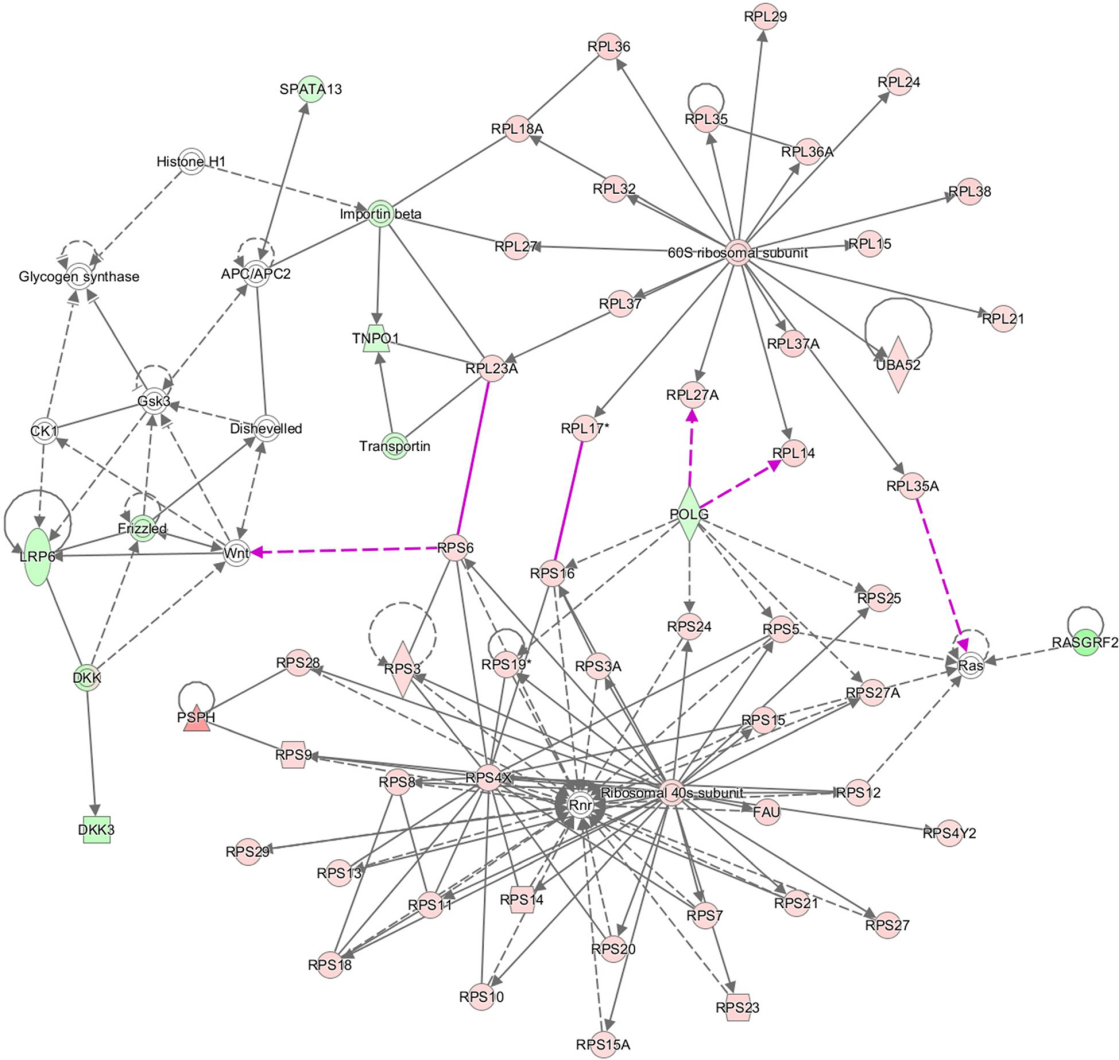
Ribosomal and protein synthesis network in hepatic tissue following dietary restriction. Merged diagram of networks 2 and 21, at the end of Period 1. Network #2: protein synthesis, RNA post-transcriptional modification. Network #21: gene expression, protein synthesis. The network is displayed graphically as nodes (genes). The node colour intensity indicates the expression of genes; with red representing up-regulation and green, down-regulation in restricted fed animals compared with ad libitum controls at the end of Period 1

Of the 1352 DEGs at the end of Period 1, and 49 DEGs at the end of Period 2, 1105 and 41 genes, respectively, were successfully mapped to a molecular or biological pathway and/or category in the IPA database. Fold changes of all statistically significant DEGs in both periods are presented in Additional file 1: Table S1 and Additional file 2: Table S2 for periods 1 and 2, respectively. Pathway analysis of these DEG lists using KEGG, revealed a number of enriched pathways. In Period 1, a total of 29 over represented enriched pathways were identified. Enriched pathways in Period 1 included those involved in cellular metabolism and ribosomal peptide synthesis (*P* < 0.0001) (Table 1). The smaller number of DEGs identified at the end of Period 2, resulted in differentially expressed genes mapping to only two pathways significantly, namely protein processing in the endoplasmic reticulum (*P* < 0.0008) and biosynthesis of unsaturated fatty acids (*P* < 0.0479). Enriched pathways of biological interest following a period of dietary restriction at the end of Period 1 are presented in Table 1. Functional enrichment analyses, corrected for multiple testing were subsequently performed on DEGs. At the end of Period 1, genes involved in processes such as protein synthesis, lipid metabolism, molecular transport, cellular growth and proliferation, cell cycle and energy production were all found to be differentially expressed (*P* < 0.05). The direction of fold change of genes identified as differentially expressed in these processes indicating an overall down-regulation of these processes, with the exception of protein synthesis which was up-regulated following a period of dietary restriction. Biological categories identified at the end of Period 1 are presented in Fig. 4. Following 55 days of re-alimentation, differentially expressed genes (*P* < 0.05) involved in processes such as cell morphology, cellular growth and proliferation as well as metabolism suggested an up-regulation of these processes during re-alimentation induced CG. Details of these biological categories in addition to others identified are presented in Fig. 5. Further details of the genes involved in some of these processes are further described in Tables 2, 3 and 4 (Table 2: nutrient transport, Table 3: cell cycle and Table 4: cellular growth and proliferation). Using IPA, a total of 25 gene networks were identified at the end of Period 1, with 7 networks identified at the end of Period 2. Details of the different networks are outlined in Additional file 3: Table S3 and Additional file 4: Table S4 for periods 1 and 2, respectively. Particular networks of interest included protein synthesis and RNA post-transcriptional modification (network 2) and gene expression and protein synthesis (network 21) in Period 1, which comprised of genes involved in protein synthesis, gene expression and cellular growth (Fig. 6).

**Table 1:** Kyoto Encyclopaedia of Genes and Genomes (KEGG) pathways that were significantly over-represented in hepatic tissue in restricted fed animals compared with ad libitum control animals following a period of dietary restriction at the end of Period 1

Enriched KEGG pathways	Over represented *P* value
Metabolic pathways	<0.0001
Ribosome	<0.0001
Steroid hormone biosynthesis	0.0007
Arginine and proline metabolism	0.0013
Glycine, serine and threonine metabolism	0.0029
Citrate cycle	0.003
Starch and sucrose metabolism	0.0035
PPAR^1^ signalling pathway	0.0085
Protein processing in endoplasmic reticulum	0.010
Tryptophan metabolism	0.0212
MAPK signalling pathway	0.028
Valine, leucine and isoleucine degradation	0.029
Tyrosine metabolism	0.036
Insulin signalling pathway	0.0498

^1^ Peroxisome proliferator-activated receptor

**Table 2:** Differentially expressed hepatic genes involved in nutrient transport in RES compared with ADLIB animals at the end of Period 1

Gene symbol	Gene name	Fold change^1^	*P* value
**Amino acid**			
*SLC36A1*	Solute carrier family 36 (proton/amino acid symporter), member 1	−1.581	0.00118
*SLC38A2*	Solute carrier family 38, member 2	1.439	0.00556
*SLC38A4*	Solute carrier family 38, member 4	−1.433	0.00312
*SLC6A14*	Solute carrier family 6 (amino acid transporter), member 14	1.541	0.016
*SLC7A2*	Solute carrier family 7 (cationic amino acid transporter, y + system), member 2	−2.853	*P* < 0.001
*SLC7A9*	Solute carrier family 7 (amino acid transporter light chain, bo,+ system), member 9	−1.427	0.0043
**Lipid**			
*SLC27A4*	Solute carrier family 27 (fatty acid transporter), member 4	−2.112	*P* < 0.001
**Carbohydrate**			
*SLC2A5*	Solute carrier family 2 (facilitated glucose/fructose transporter), member 5	−9.346	*P* < 0.001
*SLC5A1*	Solute carrier family 5 (sodium/glucose cotransporter), member 1	−1.852	0.00003
*SLC37A4*	Solute carrier family 37 (glucose-6-phosphate transporter), member 4	−1.328	0.0000892
**Mineral**			
*SLC30A10*	Solute carrier family 30, member 10	−1.677	0.000127
*SLC30A6*	Solute carrier family 30 (zinc transporter), member 6	−1.317	0.00204
*SLC41A2*	Solute carrier family 41 (magnesium transporter), member 2	−1.489	0.000712

^1^ Fold changes are up or down in restricted fed animals compared with ad libitum control animals

**Table 3:** Hepatic genes involved in the cell cycle differentially expressed following a period of dietary restriction (Period 1) and a subsequent period of re-alimentation and compensatory growth (Period 2)

Gene symbol	Gene name	Fold change^1^	*P* value
**Period 1**			
*CABLES1*	Cdk5 and Abl enzyme substrate 1	−1.589	0.00031
*CDC7* *CCND3*	Cell division cycle 7 Cyclin D3	−1.251 1.297	0.0108 0.00309
*CCNG2*	Cyclin G2	−1.488	0.000608
*CDK11A1*	Cyclin-dependent kinase 11A	−1.269	9.79E-05
*CDK12*	Cyclin-dependent kinase 12	−1.302	0.000723
*CDK2AP1*	Cyclin-dependent kinase 2 associated protein 1	1.379	0.0000513
*CDK2AP2*	Cyclin-dependent kinase 2 associated protein 2	1.370	0.00264
*CDKN1B*	Cyclin-dependent kinase inhibitor 1B (p27, Kip1)	1.328	0.0000147
*CKS2*	CDC28 protein kinase regulatory subunit 2	−2.020	0.000000000058
*DDIT3*	DNA-damage-inducible transcript 3	1.681	0.00000563
*GADD45GIP1*	Growth arrest and DNA-damage-inducible, gamma interacting protein 1	1.446	0.00019
*LZTS2*	Leucine zipper, putative tumor suppressor 2	1.372	0.00361
*NEK9*	NIMA-related kinase 9	−1.329	0.000193
*NUF2*	NUF2, NDC80 kinetochore complex component	−3.728	0.000000216
**Period 2**			
*DDIT3*	DNA-damage-inducible transcript 3	−1.976	0.0000513

^1^ Fold changes are up or down in restricted fed animals compared with ad libitum control animals

**Table 4:** Hepatic genes involved in cell growth and proliferation differentially expressed following a period of dietary restriction (Period 1) and a subsequent period of re-alimentation and compensatory growth (Period 2)

Gene symbol	Gene name	Fold change^1^	*P* value
**Period 1**			
*DYRK1A*	Dual-specificity tyrosine-(Y)-phosphorylation regulated kinase 1A	−1.262	0.00276
*DYRK1B*	Dual-specificity tyrosine-(Y)-phosphorylation regulated kinase 1B	−1.360	0.00120
*EGFR*	Epidermal growth factor receptor	−1.428	0.0105
*FGFR4*	Fibroblast growth factor receptor 4	−1.349	0.000413
*ID2*	Inhibitor of DNA binding 2, dominant negative helix-loop-helix protein	1.487	0.000464
*INHBC*	Inhibin, beta C	−1.759	0.000267
*MANF*	Mesencephalic astrocyte-derived neurotrophic factor	1.659	0.000717
*PIK3C2G*	Phosphatidylinositol-4-phosphate 3-kinase, catalytic subunit type 2 gamma	−1.293	0.0014
*ZNF516*	Zinc finger protein 516	−1.824	0.000657
**Period 2**			
*MANF*	Mesencephalic astrocyte-derived neurotrophic factor	−2.363	0.000127
*SPARC*	Secreted protein, acidic, cysteine-rich (osteonectin)	2.115	0.000257

^1^ Fold changes are up or down in restricted fed animals compared with ad libitum control animals

### qRT-PCR validation of RNAseq data

The RNAseq dataset was validated against expression data of component genes of the somatotropic axis previously examined [22] using the same RNA samples as used in the current study. Of the 12 genes evaluated at each time-point, results were consistent between methodologies for direction and magnitude of differential gene expression among genes analysed (Table 5). There was a significant (*P* < 0.05) correlation in the measurement of gene expression between the two technologies for 16 of the genes examined (over the two time-points), with a tendency towards a significant correlation (*P* < 0.1) evident for the remaining genes. Seven of the genes at the end of Period 1 and ten genes at the end of Period 2 were not detected as significantly differentially expressed in either RNAseq or qRT-PCR datasets. Five and two genes were found to be differentially expressed in both RNAseq and qRT-PCR datasets at the end of Periods 1 and 2 respectively. Details of fold changes and *P* values between RNAseq and qRT-PCR for all genes examined are presented in Table 5.

**Table 5:** Genes validated between qRT-PCR and RNAseq methodologies, including fold change (FC), P-values and correlation coefficients (R)

	RNAseq		RT-qPCR		Correlation	
Gene	FC	*P-*Value	FC	*P-*Value	R	*P-*Value
**Period 1**						
*SOCS3*	−1.17	0.244	−0.61	0.0924	0.59	0.052
*JAK2*	1.15	0.227	0.71	0.602	0.61	0.044
*STAT5B*	−1.07	0.576	−0.69	1	0.57	0.031
*IGF1*	−2.53	<.0001	−1.06	0.001	0.48	0.013
*IGFBP1*	3.62	<.0001	3.91	<.0001	0.95	<0.001
*IGFBP2*	29.25	<.0001	8.45	<.0001	0.52	0.008
*IGFBP3*	−1.11	0.139	−0.84	0.684	0.63	0.045
*IGFBP4*	1.01	0.952	0.65	0.872	0.51	0.094
*IGFBP5*	−1.48	0.401	−1.13	0.999	0.70	0.012
*IGFBP6*	1.83	<.0001	1.74	<.0001	0.65	0.023
*ALS*	1.05	0.641	1.02	0.648	0.82	0.037
*GHR*	−1.89	<.0001	−1.39	0.0274	0.77	0.048
**Period 2**						
*SOCS3*	1.05	0.722	0.065	0.963	0.49	0.089
*JAK2*	1.13	0.339	0.75	0.641	0.63	0.054
*STAT5B*	1.09	0.466	0.62	0.999	0.48	0.036
*IGF1*	−1.01	0.914	−0.76	0.995	0.72	0.037
*IGFBP1*	1.29	0.032	1.00	0.043	0.81	0.002
*IGFBP2*	1.04	0.755	0.51	0.910	0.59	0.054
*IGFBP3*	1.05	0.669	0.58	0.979	0.29	0.068
*IGFBP4*	−1.04	0.731	−0.83	0.993	0.42	0.047
*IGFBP5*	1.33	0.051	1.00	0.495	0.16	0.054
*IGFBP6*	−1.23	0.625	−0.77	0.990	0.57	0.024
*ALS*	−1.01	0.955	−0.88	1	0.49	0.078
*GHR*	1.03	0.048	1.09	0.041	0.86	0.025

## Discussion

The phenomenon of CG following a period of restricted feeding has been associated with an improved efficiency and utilisation of feed, most notably through greater overall gain in body-weight for a given level of feed intake. Indeed this was observed in the animals used in the current study, whereby animals undergoing CG were more feed efficient [9]. Several studies have investigated the physiological control of restricted feeding and CG [9–13, 24–28] however, understanding of the molecular control of CG still remains to be elucidated. This is important as genes contributing to the phenomenon may represent putative biological markers for improved growth and feed efficiency potential in cattle. The liver was chosen as a target tissue of interest, as it is a highly metabolic organ and is clearly physically affected by restricted feeding and subsequent re-alimentation [9, 11]. Indeed liver tissue of the compensating animals in the current study were found to have achieved 100 % recovery following 55 days of re-alimentation, whereas overall body-weight and carcass CG indexs [3] for RES animals were 48 and 32 % respectively, during the same time.

### Metabolism and nutrient transporters

As the liver is the central organ responsible for intermediary metabolism within the body [29], it is not surprising that a reduction in dietary intake would incur a reduction in its size and metabolic activity [30]. Following digestion of food, the chemical constituents of the feed are transferred to the liver from the small intestine where they are further metabolised. The liver can subsequently orchestrate flux and inter-conversion of nutrients and metabolites to support the changes in demand and supply of nutrients during periods of restrictive feeding. This may be controlled through alterations in gene expression, enzyme activities and the resultant nutrient fluxes which are essential for optimal liver function and nutrient inter-conversion [31, 32]. Furthermore, as a highly metabolic organ, the liver has a substantial basal energy demand, responsible for between 18 and 25 % of the total oxygen consumption in cattle [7]. In order to utilise energy more effectively and efficiently during restricted feeding, the liver has the capacity to regulate its size and metabolic activity so as to reduce energy requirements during times of limited nutrient availability [8]. Indeed we and others have shown that liver size is reduced during periods of restricted feeding [9, 11, 13]. Furthermore, in the current study we observed genes associated with amino acid, lipid and carbohydrate metabolism to be differentially expressed, with a large number of these genes down-regulated following a period of dietary restriction at the end of Period 1 (Fig. 4). Following 55 days of re-alimentation, lipid and carbohydrate metabolic processes were significantly affected by re-alimentation, with the majority of DEGs involved in both lipid and carbohydrate metabolism up-regulated in RES compared with ADLIB. These results indicate an acquired greater capacity for hepatic metabolic processes during CG, which is not surprising given the documented greater feed intake per unit of bodyweight during re-alimentation which was evident in the animals used in the current study as outlined by Keogh et al. [9]. Indeed, Burrin et al. [33] observed that metabolic processes associated with dietary energy intake were enhanced during CG in sheep. This was particularly evident in that study through a comparison of liver energy consumption in rams undergoing either maintenance (22 % energy consumption of whole body energy use) or CG (41 % energy consumption of whole body energy use).

This lower nutrient requirement of the liver to process nutrients was evidenced in the current study through down-regulation in the expression of a number of nutrient transporter genes in animals following feed restriction compared with their ad libitum fed contemporaries. Nutrient flux through the plasma membrane is facilitated by nutrient transporters. These trans-membrane proteins are substrate specific and are differentially expressed between different tissues to aid in the partitioning of nutrients. Following a period of feed restriction, genes associated with the transport of nutrients such as lipids, amino acids and carbohydrates as well as mineral transporters were differentially expressed. All DEGs related to nutrient transport displayed reduced expression at the end of Period 1 in RES animals compared with ADLIB animals, with the exception of *SLC38A2* and *SLC6A14*. Expression of *SLC6A14* has previously been shown to be up-regulated in the duodenal epithelia of cows which displayed greater feed and production efficiencies [34]. A reduction in hepatic expression of nutrient transporters following restricted feed intake has also been observed in chickens [35]. Additionally, alterations in solute carrier transporters was observed in dairy cows during negative energy balance, a period of time after calving when energy consumption is typically less than requirements [36]. Amino acid, sugar and mineral transporters were down regulated due to alterations in energy partitioning during the early post-partum period in that study, reinforcing the role of transporters in energy partitioning in highly metabolic organs such as in the liver.

### Cellular proliferation and growth

Effects of plane of nutrition on liver size and growth may be due to alterations in cellular proliferation in addition to an overall metabolic activity or workload [13, 14, 25]. We observed a number of growth and proliferative genes to be differentially expressed between feed restricted and non-restricted animals. Of note, all of these genes had lower transcript abundance in the feed restricted animals. These included genes encoding cell receptors involved in cellular growth including epidermal growth factor receptor (*EGFR*) [37] and fibroblast growth factor receptor (*FGFR4*) [38]. The transcription factor *ZNF516* which is involved in cell proliferation was also down regulated in RES animals at the end of Period 1. Signalling processes involved in cellular proliferation and growth such as the dual specificity tyrosine phosphorylation regulated kinases *DYRK1A* and *DYRK1B* [39] and PI3-kinase signalling including *PIK3C2G* [40] all had reduced expression at the end of a period of restricted feeding. The TGF-beta superfamily is involved in many cellular processes including cell growth and differentiation [41]. A gene which codes for the beta C chain of inhibin (*INHBC)*, which is a member of this superfamily, displayed reduced expression in the feed restricted animals. Additionally, there was up-regulation of genes associated with the inhibition of cellular proliferation, including: *MANF* and *ID2.* A previous examination of liver gene expression using microarray technology also showed alterations in genes associated with cellular growth and proliferation in hepatic tissue of steers following restricted feeding [14]. However, there was no consistency in the specific genes identified between the two studies. This may be due to differences in experimental design between the two studies in addition to utilising varying technologies, or alternatively, it may be due to sampling tissue at different time points, or stringency in data analysis, ultimately confounding comparison of the outcomes. However, in that study, the authors subsequently noted greater expression of hepatic genes associated with cellular proliferation and growth in compensating animals early into re-alimentation (days 1 and 14 of re-alimentation). A similar finding was observed in our study with cell cycle and growth processes up-regulated at the end of Period 1 (Fig. 4). One gene in particular *SPARC,* which codes for a cysteine-rich acidic matrix-associated protein, was up-regulated in both hepatic tissue studies, during the initial stages of accelerated growth [14] and also following 55 days of re-alimentation in the current study. This gene appears to regulate cell growth through interactions with the extracellular matrix and cytokines [42]. *SPARC* may be a potential genomic target for enhanced CG or improved feed efficiency potential in cattle particularly as it was differentially expressed during both the initiation of re-alimentation [14] and also during more sustained CG in the current study.

Continued increased expression of cellular proliferation genes by 55 days into the re-alimentation period was somewhat unexpected as by then, at least on a weight recovery basis, the liver appeared to have compensated fully (displayed a 100 % weight recovery index). However, as only 2 genes associated with proliferation and growth were up-regulated by day 55 of re-alimentation compared with a larger number observed earlier into re-alimentation in other studies [14], this suggests that the accelerated growth of this organ had declined by day 55 of re-alimentation which is consistent with overall recovery of this tissue. However, it must be noted that although no difference was apparent in the weight of the liver between treatment groups at the end of Period 2, a return to equal mass of the liver may not reflect a return to equal function. Thus, further evaluations on the functional control of hepatic tissue during CG is warranted.

### Cell cycle

Genes involved in the cell cycle were also differentially expressed in liver of animals undergoing feed restriction and subsequent compensation, relative to their ad libitum fed contemporaries. Genes coding for proteins important to the G1/S transition of the cell cycle were also down regulated in RES animals. For example, *NEK9,* which is a regulator of mitotic progression, participating in the control of spindle dynamics and chromosome separation [43] was down regulated in RES animals. Additionally, *CDC7* which encodes a cell division cycle protein with kinase activity that phosphorylates critical substrates regulating the G1/S phase transition [44]. Down regulation of genes involved in the G1/S transition occurred in parallel with up regulation of *GADD45GIP1*- a nuclear-localised protein that may be induced by p53 and regulates the cell cycle by inhibiting G1 to S phase progression [45]. The encoded protein acts as a negative regulator of G1 to S phase progression by inhibiting cyclin-dependent kinases.

Genes coding for structural components of the cell cycle were also affected by the feed restriction regimen employed here. For example, *NUF2* which codes for a component of the essential kinetochore-associated NDC80 complex, which is required for chromosome segregation and spindle checkpoint activity was down regulated in the restricted animals. The protein encoded by this gene is required for kinetochore integrity and the organisation of stable microtubule binding sites in the outer plate of the kinetochore [46]. The effect of restricted feeding on hepatic cell cycle progression was further established through up-regulation of genes associated with cell cycle inhibition including, *DDIT3* and *LZTS2. DDIT3*, a transcription factor that induces cell cycle arrest and apoptosis [47], was up regulated in feed restricted animals at the end of Period 1. Differences in cell cycle genes followed a similar pattern to those involved in cellular proliferation. In particular, with respect to the up regulation of proliferative genes 55 days into re-alimentation, where *DDIT3*, was subsequently down regulated in animals undergoing CG.

Cyclins are a family of proteins that control the progression of the cell cycle by activating cyclin-dependent kinase enzymes [48]. Cyclin G2 (*CCNG2*), cyclin-dependent kinase 11a (*CDK11A*), cyclin-dependent kinase 12 (*CKS2*) and a cyclin regulatory subunit (*CDK12*) were all down regulated in restricted animals following 125 days of feed restriction. Additionally, *CABLES1*, which encodes a protein involved in regulating the cell cycle through interactions with cyclin-dependent kinases was also down-regulated in RES animals at the end of Period 1. Down regulation of these cell cycle progression genes further implies less cell cycle division and replication taking place in hepatic tissue of the restricted animals compared with the ad libitum fed control animals. However, *CCDN3* which forms a complex with, and functions as a regulatory subunit of cyclin dependent kinase 4 and 6, whose activity is required for the G1/S cell cycle transition, was in fact up-regulated in animals following a period of restricted feeding. This result suggests that the *GADD45GIP1* gene was in fact expressed in order to cause a disruption to the G1/S phase transition in the cell cycle. Indeed inhibitors of cyclin activity were identified as differentially expressed following a period of restricted feeding. Proteins encoded by *CDK2AP1* and *CDK2AP2* are both thought to function as negative regulators of cyclin dependent kinase 2, during S phase of the cell cycle [49]. Similarly, *CDKN1B* which encodes a cyclin-dependent kinase inhibitor was also up regulated in animals fed a restricted diet. The encoded protein binds to and prevents the activation of cyclin E-CDK2 or cyclin D-CDK4 complexes and thus controls the cell cycle progression at G1 and is ultimately involved in G1 arrest. Overall, it is apparent that the documented difference in liver weight and volume at the end of a restricted feeding regimen may have been due to a reduction in the occurrence of hepatic cell division. As only one gene associated with cell division was differentially expressed 55 days into re-alimentation, together with the observed full recovery of liver weight, suggests that most, if not all, of hepatic tissue compensation had occurred at this time.

### Protein synthesis

Perhaps the most striking result from this dataset was the large number of DEGs with denoted ribosomal functions (Additional file 1: Table S1). This was also evident through KEGG pathway analysis, where at the end of Period 1, the ribosome was identified as the second most significantly over-represented pathway (Table 1). The ribosome is a large and complex molecular machine that serves as the primary site of biological protein synthesis [50]. This organelle works to link amino acids together in the order specified by messenger RNA molecules and consists of two components, the small ribosomal subunit (40S) which reads the RNA, and the large subunit (60S) which joins amino acids to form a polypeptide chain [50]. During differential feeding in Period 1, 28 genes coding for components of the ribosomal 40S subunit and 35 genes of the 60S ribosome were all up-regulated (Additional file 1: Table S1). Greater expression of these genes in Period 1 coincided with greater expression of a number of genes involved in amino acid synthesis and protein processing. Additionally, a number of tRNA (transfer RNA) genes were also up regulated following a period of restricted feeding. Amino acids are selected, collected and carried to the ribosome by tRNA, which enter the ribosome and bind to the mRNA chain. Increased protein synthesis has been documented previously in rodents when examining the effect of feed restriction on ageing properties [51–53]. Greater expression of genes related to protein synthesis in RES animals suggests a greater efficiency and utilisation of diet derived nutrients in hepatic tissue in these animals during dietary restriction.

A greater degree of protein deposition occurs within the body during the initial stages of CG [54–56]. This is thought to act through a necessity to increase the metabolic capacity of organs such as the liver and gastrointestinal tract in order to be able to process the greater quantities of nutrients available during re-alimentation. There is potential, given the up-regulation of hepatic genes associated with protein synthesis during restricted feeding, that this may continue on into the re-alimentation period and ultimately contribute to the occurrence of CG through repletion of metabolically important tissues. In their examination of hepatic DEGs, Connor et al. [14] observed up-regulation of ribosomal genes on the first day of re-alimentation. Of the ribosomal genes identified in that study, 19 genes coding for subunits of the large ribosome, whilst 15 encoded subunits of the small 40S subunit were up-regulated in animals undergoing CG on the first day of re-alimentation. In our own study we identified 14 60S ribosomal genes which were in agreement with the findings of Connor et al. [14], whilst 14 40S ribosomal genes were also common between the two studies. By day 55 of re-alimentation, only one ribosomal gene, *RPS27,* was differentially expressed between RES and ADLIB animals and this was down-regulated in RES. Taken together with evidence presented earlier, it is appropriate to suggest that hepatic compensation was complete at this stage of re-alimentation. When metabolically important tissues have been restored fully, there is an apparent increase in adipose deposition as opposed to protein tissue deposition [55, 57–59]. An indication towards an increase in adipose deposition occurring during CG was obtained through up-regulation of genes involved in adipose deposition including *FADS1* and *SREBF1*on day 55 of re-alimentation. These results potentially indicate an increase in adipose deposition together with a decrease in protein deposition in hepatic tissue coinciding with a decrease in overall body growth rate at the end of Period 2.

### Potential molecular biomarkers

The CG phenomenon is utilised in beef production systems worldwide [60, 61]. However knowledge of the underlying molecular control regulating the expression of CG is lacking. A greater understanding of the genetic basis for CG is critical to the future effective exploitation of the trait and may lead to the discovery of DNA-based biomarkers which could be incorporated into genomic selection breeding programmes to select animals with a greater propensity to display CG following prior dietary restriction. Furthermore, as CG is associated with an improvement in feed efficiency, differentially expressed genes identified in this study may contribute to breeding protocols for the selection of animals with improved feed efficiency. An examination of the findings of both the current study and those of Connor et al. [14], show that a number of DEGs were observed to be in agreement during restricted feeding in the current dataset and during early CG [14] including: *AKR1C3; INSIG1; SELK* and *UBL5*. These genes may be potential targets for the accelerated growth observed during the early stages of CG. Additionally, hepatically expressed genes which had greater transcript abundance in feed efficient (low residual feed intake) animals including *GOLTA1, IDH2, INHBA, PSPH, PYCR1, RPS4X* and *STEAP4* [62] were also up-regulated in the present study during restricted feeding and may represent markers for feed efficiency. During CG, *SPARC* was up-regulated in both the current study and the data of Connor et al. [14]. Only one common gene between these two studies during the CG phase may be due to different sampling time points, with samples in the current study taken on day 55 of re-alimentation and samples taken by Connor et al. [14] on the first day of re-alimentation. Additionally, the gene *SREBF1* was identified as up-regulated in the current hepatic data-set on day 55 of re-alimentation, as well as on day 15 of re-alimentation in skeletal muscle in the same animals undergoing CG as in the current study [63]. *SREBF1* codes for a sterol regulator element-binding transcription factor and has a crucial role in energy homeostasis through promotion of glycolysis, lipogenesis and adipogenesis [64–66]. Further investigation is warranted to determine if these results can be utilised as potential molecular markers for CG and feed efficiency in cattle.

## Conclusions

During dietary restriction evidence of reduced metabolic activity of the liver was apparent through less mRNA abundance of nutrient transporters. Additionally, through an examination of differential transcript abundance we observed evidence for a reduction in cell cycle processes as well as a reduction in cellular proliferation and growth. For both processes this was manifested as a down-regulation in the expression of promoter genes coincident with an increase in transcripts coding for inhibitor proteins. Our data also suggests that an increased capacity for protein synthesis following feed restriction may sustain into CG in re-alimentation. This may then allow for the preferential deposition of protein ahead of adipose deposition in order to aid in the recovery of metabolically active organs such as the liver and gastrointestinal tract, which are required to facilitate the increased feed intake and nutrient availability associated with re-alimentation. Our results further suggest that hepatic protein synthesis may have subsided by 55 days into re-alimentation, in favour of adipose deposition and may signal full hepatic tissue recovery at that stage. In the context of the current study, 55 days into re-alimentation may have been too late to identify large differences in pathways and genes regulating CG, as although an overall body compensatory index of only 48 % was achieved in this time, the liver had fully recovered by day 55. Potentially sampling hepatic tissue earlier into re-alimentation for example following 1 month of re-alimentation may yield further information on the underlying biological control regulating the expression of CG. However, genes differentially expressed by day 55 in the current study may represent a more sustained or prolonged CG. It must be noted, however, that a return to equal mass of the liver following 55 days of re-alimentation and CG may not reflect a return to equal function, thus, further evaluations on the functional control of hepatic tissue during CG is warranted. The new knowledge generated in this study offer further insights into some of the molecular processes underlying restricted and CG in cattle. Furthermore, differential gene expression patterns provide data which may be further integrated and used for the selection of robust biomarkers to identify animals with superior genetic potential for CG and feed efficiency.

## Additional files

Additional file 1: Table S1. Differentially expressed genes following a period of restricted feeding. (DOCX 120 kb) Additional file 2: Table S2. Differentially expressed genes following a period of compensatory growth in re-alimentation. (DOCX 17 kb) Additional file 3: Table S3. Networks generated from gene expression data of restricted versus ad libitum fed bulls by IPA. (DOCX 21 kb) Additional file 4: Table S4. Networks generated from gene expression data of bulls undergoing compensatory growth versus ad libitum fed bulls by IPA. (DOCX 15 kb)
