# Supplementary material for: Effect of dietary restriction and subsequent re-alimentation on the transcriptional profile of hepatic tissue in cattle
Source: BMC Genomics. 2016 Mar 17;17:244. doi: 10.1186/s12864-016-2578-5 (PMC4794862; doi:10.1186/s12864-016-2578-5)
Supplement: Additional file 1: Table S1. — Differentially expressed genes following a period of restricted feeding. (DOCX 120 kb) [file 12864_2016_2578_MOESM1_ESM.docx]

**Additional file 1: Table S1.** Differentially expressed genes following a period of restricted feeding

| Symbol | Entrez Gene Name | Fold Change^1^ | p-value |
| --- | --- | --- | --- |
| *AADAC* | Arylacetamide deacetylase | -1.477 | 0.00054 |
| *AADAT* | Aminoadipate aminotransferase | -1.366 | 0.0006 |
| *AAMDC* | Adipogenesis associated, Mth938 domain containing | 1.434 | 2.3E-05 |
| *AASS* | Aminoadipate-semialdehyde synthase | -2.049 | 7.22E-12 |
| *ABAT* | 4-aminobutyrate aminotransferase | -1.339 | 0.00205 |
| *ABCA5* | ATP-binding cassette, sub-family A (ABC1), member 5 | -1.33 | 0.00121 |
| *ABCB10* | ATP-binding cassette, sub-family B (MDR/TAP), member 10 | -1.688 | 4.77E-08 |
| *ABCB11* | ATP-binding cassette, sub-family B (MDR/TAP), member 11 | -1.553 | 0.00076 |
| *ABCC2* | ATP-binding cassette, sub-family C (CFTR/MRP), member 2 | -1.444 | 0.00407 |
| *ABCC6* | ATP-binding cassette, sub-family C (CFTR/MRP), member 6 | -1.844 | 4.84E-10 |
| *ABCD1* | ATP-binding cassette, sub-family D (ALD), member 1 | -1.408 | 0.00037 |
| *ABHD1* | Abhydrolase domain containing 1 | 1.91 | 1.72E-07 |
| *ABHD15* | Abhydrolase domain containing 15 | -1.586 | 0.00012 |
| *ACAP1* | ArfGAP with coiled-coil, ankyrin repeat and PH domains 1 | 1.609 | 0.00034 |
| *ACE2* | Angiotensin I converting enzyme 2 | -7.421 | 1.63E-19 |
| *ACMSD* | Aminocarboxymuconate semialdehyde decarboxylase | -2.275 | 5.03E-13 |
| *ACOT8* | Acyl-CoA thioesterase 8 | 1.333 | 0.00248 |
| *ACOX1* | Acyl-CoA oxidase 1, palmitoyl | -1.376 | 0.00248 |
| *ACP5* | Acid phosphatase 5, tartrate resistant | 1.691 | 0.00044 |
| *ACSM1* | Acyl-CoA synthetase medium-chain family member 1 | -1.403 | 0.00135 |
| *ACSM2A* | Acyl-CoA synthetase medium-chain family member 2A | -1.587 | 2.26E-06 |
| *ACSM5* | Acyl-CoA synthetase medium-chain family member 5 | -1.653 | 7.19E-06 |
| *ACVR1* | Activin A receptor, type I | 1.251 | 0.00115 |
| *ADAMTS10* | ADAM metallopeptidase with thrombospondin type 1 motif, 10 | 1.765 | 0.00094 |
| *ADAMTSL4* | ADAMTS-like 4 | 1.504 | 0.00115 |
| *ADAR* | Adenosine deaminase, RNA-specific | -1.333 | 0.00015 |
| *ADH4* | Alcohol dehydrogenase 4 (class II), pi polypeptide | -1.338 | 0.00114 |
| *ADIRF* | Adipogenesis regulatory factor | 2.254 | 6.12E-06 |
| *AFF1* | AF4/FMR2 family, member 1 | -1.497 | 1.73E-06 |
| *AFMID* | Arylformamidase | -1.369 | 0.00011 |
| *AGL* | Amylo-alpha-1, 6-glucosidase, 4-alpha-glucanotransferase | -1.48 | 0.00023 |
| *AGPAT9* | 1-acylglycerol-3-phosphate O-acyltransferase 9 | 1.991 | 5.65E-08 |
| *AIFM2* | Apoptosis-inducing factor, mitochondrion-associated, 2 | 1.308 | 0.002 |
| *AIM1* | Absent in melanoma 1 | -1.433 | 0.00016 |
| *AK4* | Adenylate kinase 4 | 2.105 | 1.70E-06 |
| *AKNA* | AT-hook transcription factor | 1.419 | 0.00396 |
| *AKR1C3* | Aldo-keto reductase family 1, member C3 | 2.213 | 0.00286 |
| *AKR1D1* | Aldo-keto reductase family 1, member D1 | -1.483 | 0.00017 |
| *AKR7A2* | Aldo-keto reductase family 7, member A2 (aflatoxin aldehyde reductase) | 1.412 | 0.00055 |
| *ALAD* | Aminolevulinate dehydratase | 1.411 | 1.5E-05 |
| *ALCAM* | Activated leukocyte cell adhesion molecule | -1.512 | 2.16E-05 |
| *ALDH1L2* | Aldehyde dehydrogenase 1 family, member L2 | 3.244 | 1.33E-09 |
| *ALDH4A1* | Aldehyde dehydrogenase 4 family, member A1 | -1.696 | 7.30E-06 |
| *ALDH5A1* | Aldehyde dehydrogenase 5 family, member A1 | -1.647 | 0.00032 |
| *ALDH6A1* | Aldehyde dehydrogenase 6 family, member A1 | -1.384 | 0.00215 |
| *ALDOB* | Aldolase B, fructose-bisphosphate | -1.781 | 5.83E-05 |
| *AMDHD1* | Amidohydrolase domain containing 1 | -1.498 | 0.0001 |
| *AMFR* | Autocrine motility factor receptor, E3 ubiquitin protein ligase | -1.359 | 0.0025 |
| *AMIGO2* | Adhesion molecule with Ig-like domain 2 | -2.046 | 6.53E-06 |
| *AMT* | Aminomethyltransferase | 1.33 | 0.00028 |
| *AMY2A* | Amylase, alpha 2A (pancreatic) | -1.354 | 0.00541 |
| *ANGPTL4* | Angiopoietin-like 4 | 2.137 | 1.1E-05 |
| *ANKH* | ANKH inorganic pyrophosphate transport regulator | -1.525 | 4.62E-05 |
| *ANKRD44* | Ankyrin repeat domain 44 | -1.402 | 0.00052 |
| *ANP32B* | Acidic (leucine-rich) nuclear phosphoprotein 32 family, member B | 1.273 | 0.00026 |
| *ANPEP* | Alanyl (membrane) aminopeptidase | 3.058 | 3.18E-17 |
| *ANTXR2* | Anthrax toxin receptor 2 | -1.261 | 0.00421 |
| *AP1G1* | Adaptor-related protein complex 1, gamma 1 subunit | -1.429 | 0.0002 |
| *AP5B1* | Adaptor-related protein complex 5, beta 1 subunit | 1.363 | 0.00461 |
| *APBB3* | Amyloid beta (A4) precursor protein-binding, family B, member 3 | 1.435 | 0.00134 |
| *APCDD1* | Adenomatosis polyposis coli down-regulated 1 | 1.357 | 0.00044 |
| *APIP* | APAF1 interacting protein | 1.524 | 2.44E-06 |
| *APOA1* | Apolipoprotein A-I | 1.692 | 0.00326 |
| *APOA4* | Apolipoprotein A-IV | 3.158 | 0.00032 |
| *APOA5* | Apolipoprotein A-V | -1.548 | 6.59E-06 |
| *APOC4* | Apolipoprotein C-IV | 1.304 | 0.00236 |
| *APOF* | Apolipoprotein F | -1.572 | 7.78E-06 |
| *APOL2* | Apolipoprotein L, 2 | -1.681 | 0.00214 |
| *App* | Amyloid beta (A4) precursor protein | 1.466 | 7.61E-06 |
| *AREL1* | Apoptosis resistant E3 ubiquitin protein ligase 1 | -1.583 | 1.77E-05 |
| *ARFGEF2* | ADP-ribosylation factor guanine nucleotide-exchange factor 2 (brefeldin A-inhibited) | -1.407 | 0.00131 |
| *ARG1* | Arginase 1 | -1.662 | 2.17E-05 |
| *ARG2* | Arginase 2 | -2.363 | 5.51E-09 |
| *ARHGAP35* | Rho GTPase activating protein 35 | -1.339 | 0.00062 |
| *ARHGEF12* | Rho guanine nucleotide exchange factor (GEF) 12 | -1.317 | 0.00195 |
| *ARHGEF19* | Rho guanine nucleotide exchange factor (GEF) 19 | -1.399 | 0.00124 |
| *ARHGEF37* | Rho guanine nucleotide exchange factor (GEF) 37 | -1.942 | 0.00014 |
| *ARHGEF38* | Rho guanine nucleotide exchange factor (GEF) 38 | -1.79 | 0.0009 |
| *ARL4D* | ADP-ribosylation factor-like 4D | -1.578 | 1.16E-05 |
| *ARSA* | Arylsulfatase A | 1.524 | 6.17E-05 |
| *ARSG* | Arylsulfatase G | -1.357 | 0.00413 |
| *ASAP3* | ArfGAP with SH3 domain, ankyrin repeat and PH domain 3 | -1.708 | 1.34E-06 |
| *ASB11* | Ankyrin repeat and SOCS box containing 11 | 1.267 | 0.00077 |
| *ASB9* | Ankyrin repeat and SOCS box containing 9 | -2.339 | 0.00033 |
| *ASCC3* | Activating signal cointegrator 1 complex subunit 3 | -1.313 | 0.0003 |
| *ASNS* | Asparagine synthetase (glutamine-hydrolyzing) | 2.989 | 3.21E-15 |
| *ASPG* | Asparaginase homolog (S. cerevisiae) | -4.626 | 1.83E-34 |
| *ASPSCR1* | Alveolar soft part sarcoma chromosome region, candidate 1 | 1.304 | 0.0011 |
| *ATAD3A* | ATPase family, AAA domain containing 3A | 1.324 | 0.00056 |
| *ATF3* | Activating transcription factor 3 | 1.543 | 0.00454 |
| *ATF4* | Activating transcription factor 4 | 1.614 | 1.29E-07 |
| *ATF5* | Activating transcription factor 5 | 2.568 | 4.43E-09 |
| *ATG2A* | Autophagy related 2A | -1.388 | 0.00498 |
| *ATL2* | Atlastin GTPase 2 | -1.346 | 0.00011 |
| *ATP11B* | ATPase, class VI, type 11B | -1.389 | 0.00046 |
| *ATP2B2* | ATPase, Ca++ transporting, plasma membrane 2 | -1.654 | 7.17E-06 |
| *ATP2C1* | ATPase, Ca++ transporting, type 2C, member 1 | -1.288 | 0.0012 |
| *ATP5S* | ATP synthase, H+ transporting, mitochondrial Fo complex, subunit s (factor B) | 1.359 | 0.00127 |
| *ATP6V0A1* | ATPase, H+ transporting, lysosomal V0 subunit a1 | -1.316 | 0.00034 |
| *ATP6V0E2* | ATPase, H+ transporting V0 subunit e2 | -1.314 | 0.00403 |
| *ATP8B1* | ATPase, aminophospholipid transporter, class I, type 8B, member 1 | -1.509 | 2.74E-05 |
| *ATP9B* | ATPase, class II, type 9B | -1.253 | 0.00105 |
| *ATXN1* | Ataxin 1 | -1.431 | 0.00064 |
| *AZGP1* | Alpha-2-glycoprotein 1, zinc-binding | 1.533 | 0.00365 |
| *B3GALT4* | UDP-Gal:betaGlcNAc beta 1,3-galactosyltransferase, polypeptide 4 | 1.423 | 0.00139 |
| *BBX* | Bobby sox homolog (Drosophila) | -1.385 | 0.00041 |
| *BCL2L2* | BCL2-like 2 | 1.336 | 0.00118 |
| *BCO2* | Beta-carotene oxygenase 2 | -1.327 | 0.00226 |
| *BEGAIN* | Brain-enriched guanylate kinase-associated | -3.07 | 6.09E-11 |
| *BEND5* | BEN domain containing 5 | 1.711 | 0.00105 |
| *BEX2* | Brain expressed X-linked 2 | 1.369 | 0.00047 |
| *BICC1* | Bicaudal C homolog 1 (Drosophila) | -1.437 | 0.00466 |
| *BIN3* | Bridging integrator 3 | 1.275 | 0.0015 |
| *BLMH* | Bleomycin hydrolase | -1.278 | 0.00024 |
| *BLOC1S1* | Biogenesis of lysosomal organelles complex-1, subunit 1 | 1.32 | 0.00091 |
| *BLVRB* | Biliverdin reductase B (flavin reductase (NADPH)) | 1.944 | 1.70E-09 |
| *BMP5* | Bone morphogenetic protein 5 | 1.815 | 0.0057 |
| *BRCA1* | Breast cancer 1, early onset | -1.779 | 0.00015 |
| *BRCC3* | BRCA1/BRCA2-containing complex, subunit 3 | -1.381 | 0.00029 |
| *BRMS1L* | Breast cancer metastasis-suppressor 1-like | 1.581 | 0.00103 |
| *BTG3* | BTG family, member 3 | 1.789 | 0.00011 |
| *C11orf71* | Chromosome 11 open reading frame 71 | 1.496 | 3.69E-05 |
| *C19orf12* | Chromosome 19 open reading frame 12 | 1.857 | 5.36E-09 |
| *C19orf43* | Chromosome 19 open reading frame 43 | 1.304 | 0.0024 |
| *C1GALT1* | Core 1 synthase, glycoprotein-N-acetylgalactosamine 3-beta-galactosyltransferase, 1 | -1.343 | 0.00066 |
| *C1QTNF9* | C1q and tumor necrosis factor related protein 9 | 2.055 | 0.00524 |
| *C5* | Complement component 5 | -1.487 | 0.00162 |
| *C5orf51* | Chromosome 5 open reading frame 51 | -1.35 | 0.00427 |
| *C7orf50* | Chromosome 7 open reading frame 50 | 1.453 | 0.00233 |
| *C9orf152* | Chromosome 9 open reading frame 152 | -1.464 | 0.00194 |
| *CA13* | Carbonic anhydrase XIII | -1.607 | 9.19E-05 |
| *CABLES1* | Cdk5 and Abl enzyme substrate 1 | -1.589 | 0.00031 |
| *CACHD1* | Cache domain containing 1 | -1.514 | 0.00388 |
| *CADPS2* | Ca++-dependent secretion activator 2 | -1.396 | 0.00064 |
| *Cald1* | Caldesmon 1 | -1.35 | 7.08E-05 |
| *CAMK2D* | Calcium/calmodulin-dependent protein kinase II delta | -1.277 | 0.0004 |
| *CAMK2N1* | Calcium/calmodulin-dependent protein kinase II inhibitor 1 | 1.354 | 0.0054 |
| *CAMLG* | Calcium modulating ligand | 1.258 | 0.00127 |
| *CARHSP1* | Calcium regulated heat stable protein 1, 24kDa | 1.454 | 4.37E-07 |
| *CARNS1* | Carnosine synthase 1 | -1.394 | 0.00051 |
| *CARS* | Cysteinyl-tRNA synthetase | 1.747 | 1.78E-09 |
| *CBFA2T2* | Ccore-binding factor, runt domain, alpha subunit 2; translocated to, 2 | -1.875 | 5.98E-06 |
| *CBR1* | Carbonyl reductase 1 | 1.636 | 9.51E-08 |
| *CCDC157* | Coiled-coil domain containing 157 | -1.849 | 0.00556 |
| *CCDC167* | Coiled-coil domain containing 167 | 1.468 | 2.52E-05 |
| *CCDC25* | Coiled-coil domain containing 25 | -1.343 | 0.00018 |
| *CCDC38* | Coiled-coil domain containing 38 | -4.06 | 9.14E-06 |
| *CCDC42* | Coiled-coil domain containing 42 | 1.534 | 0.00197 |
| *CCDC69* | Coiled-coil domain containing 69 | -2.961 | 7.51E-07 |
| *CCDC86* | Coiled-coil domain containing 86 | 1.465 | 0.00089 |
| *CCL5* | Chemokine (C-C motif) ligand 5 | 1.778 | 0.00192 |
| *CCND3* | Cyclin D3 | 1.297 | 0.00309 |
| *CCNG2* | Cyclin G2 | -1.488 | 0.00061 |
| *CD14* | CD14 molecule | 1.625 | 2.05E-07 |
| *CD151* | CD151 molecule (Raph blood group) | 1.386 | 0.0021 |
| *CD27* | CD27 molecule | 1.731 | 0.00197 |
| *CD276* | CD276 molecule | -1.365 | 0.00068 |
| *CD3D* | CD3d molecule, delta (CD3-TCR complex) | 1.853 | 0.00172 |
| *CD3E* | CD3e molecule, epsilon (CD3-TCR complex) | 1.788 | 0.00057 |
| *CD3G* | CD3g molecule, gamma (CD3-TCR complex) | 1.724 | 0.00245 |
| *CD47* | CD47 molecule | -1.257 | 0.00353 |
| *CD6* | CD6 molecule | 2.149 | 0.00073 |
| *CD8B* | CD8b molecule | 2.381 | 0.00148 |
| *CD99* | CD99 molecule | 1.327 | 0.00217 |
| *CDA* | Cytidine deaminase | -2.05 | 2.26E-05 |
| *CDC7* | Cell division cycle 7 | -1.251 | 0.0108 |
| *Cdca7* | Cell division cycle associated 7 | 1.641 | 0.00176 |
| *CDK11A* | Cyclin-dependent kinase 11A | -1.269 | 9.79E-05 |
| *CDK12* | Cyclin-dependent kinase 12 | -1.302 | 0.00072 |
| *CDK2AP1* | Cyclin-dependent kinase 2 associated protein 1 | 1.379 | 5.13E-05 |
| *CDK2AP2* | Cyclin-dependent kinase 2 associated protein 2 | 1.37 | 0.00264 |
| *CDKN1B* | Cyclin-dependent kinase inhibitor 1B (p27, Kip1) | 1.328 | 1.47E-05 |
| *CDO1* | Cysteine dioxygenase type 1 | -1.374 | 0.00091 |
| *CELSR2* | Cadherin, EGF LAG seven-pass G-type receptor 2 | -1.498 | 0.00041 |
| *CEP83* | Centrosomal protein 83kDa | -1.383 | 0.00119 |
| *CES2* | Carboxylesterase 2 | -1.424 | 0.00097 |
| *CFH* | Complement factor H | -1.602 | 0.00171 |
| *CFL2* | Cofilin 2 (muscle) | 1.398 | 0.00021 |
| *CGNL1* | Cingulin-like 1 | -1.337 | 0.00222 |
| *CHCHD10* | Coiled-coil-helix-coiled-coil-helix domain containing 10 | 1.457 | 0.00101 |
| *CHI3L2* | Chitinase 3-like 2 | 2.933 | 0.0008 |
| *CHPF* | Chondroitin polymerizing factor | 1.455 | 0.00326 |
| *CIRBP* | Cold inducible RNA binding protein | 1.363 | 0.0003 |
| *CITED4* | Cbp/p300-interacting transactivator, with Glu/Asp-rich carboxy-terminal domain, 4 | 1.608 | 0.00564 |
| *CKAP2* | Cytoskeleton associated protein 2 | -1.885 | 0.0022 |
| *CKB* | Creatine kinase, brain | -1.601 | 8.82E-05 |
| *CKS2* | CDC28 protein kinase regulatory subunit 2 | -2.02 | 5.75E-08 |
| *CLCN3* | Chloride channel, voltage-sensitive 3 | -1.472 | 1.54E-06 |
| *CLCN4* | Chloride channel, voltage-sensitive 4 | -1.492 | 0.0014 |
| *CLEC3B* | C-type lectin domain family 3, member B | 1.438 | 5.94E-05 |
| *CLEC4G* | C-type lectin domain family 4, member G | 1.557 | 7.25E-06 |
| *CLSTN1* | Calsyntenin 1 | -1.338 | 0.00011 |
| *CLTB* | Clathrin, light chain B | 1.261 | 0.00249 |
| *CMC1* | C-x(9)-C motif containing 1 | 1.319 | 0.00402 |
| *CMTM8* | CKLF-like MARVEL transmembrane domain containing 8 | 1.323 | 0.0002 |
| *CMYA5* | Cardiomyopathy associated 5 | -1.713 | 0.00137 |
| *CNIH4* | Cornichon family AMPA receptor auxiliary protein 4 | 1.32 | 0.00074 |
| *CNNM2* | Cyclin M2 | -1.434 | 0.00215 |
| *COL1A2* | Collagen, type I, alpha 2 | -1.803 | 0.00524 |
| *COLEC11* | Collectin sub-family member 11 | 1.469 | 0.00015 |
| *COMMD1* | Copper metabolism (Murr1) domain containing 1 | 1.378 | 5.69E-05 |
| *COX8A* | Cytochrome c oxidase subunit VIIIA (ubiquitous) | 1.31 | 0.00109 |
| *CPNE3* | Copine III | -1.396 | 8.13E-05 |
| *CPQ* | Carboxypeptidase Q | -3.399 | 0.0002 |
| *CPS1* | Carbamoyl-phosphate synthase 1, mitochondrial | -1.561 | 0.00034 |
| *CPT1A* | Carnitine palmitoyltransferase 1A (liver) | -1.354 | 0.00094 |
| *CR2* | Complement component (3d/Epstein Barr virus) receptor 2 | 1.858 | 0.00017 |
| *CRAT* | Carnitine O-acetyltransferase | -1.327 | 0.00098 |
| *CREB3L1* | cAMP responsive element binding protein 3-like 1 | 1.791 | 1.59E-06 |
| *CREB3L3* | cAMP responsive element binding protein 3-like 3 | 1.735 | 3.30E-06 |
| *CREBZF* | CREB/ATF bZIP transcription factor | 1.305 | 0.00302 |
| *CRIP1* | Cysteine-rich protein 1 (intestinal) | 1.829 | 7.66E-06 |
| *CRP* | C-reactive protein, pentraxin-related | -1.517 | 0.00038 |
| *CRY1* | Cryptochrome 1 (photolyase-like) | 1.669 | 0.00391 |
| *CRYL1* | Crystallin, lambda 1 | -1.523 | 1.88E-05 |
| *CTNNBIP1* | Catenin, beta interacting protein 1 | -1.611 | 5.56E-05 |
| *CTNS* | Cystinosin, lysosomal cystine transporter | -1.326 | 0.00051 |
| *CTPS1* | CTP synthase 1 | 1.621 | 0.00043 |
| *CTSC* | Cathepsin C | -1.625 | 0.00029 |
| *CUL4B* | Cullin 4B | -1.474 | 0.0004 |
| *CUX2* | Cut-like homeobox 2 | -1.687 | 0.00018 |
| *CXCL9* | Chemokine (C-X-C motif) ligand 9 | 1.817 | 0.00048 |
| *CYLD* | Cylindromatosis (turban tumor syndrome) | -1.531 | 3.77E-09 |
| *CYP26A1* | Cytochrome P450, family 26, subfamily A, polypeptide 1 | -1.838 | 0.00024 |
| *CYP2C19* | Cytochrome P450, family 2, subfamily C, polypeptide 19 | 4.334 | 2.95E-08 |
| *CYP2C9* | Cytochrome P450, family 2, subfamily C, polypeptide 9 | 4.334 | 2.95E-08 |
| *CYP2E1* | Cytochrome P450, family 2, subfamily E, polypeptide 1 | 2.663 | 0.00013 |
| *CYP2J2* | Cytochrome P450, family 2, subfamily J, polypeptide 2 | -1.348 | 0.0006 |
| *CYP39A1* | Cytochrome P450, family 39, subfamily A, polypeptide 1 | -1.708 | 5.15E-10 |
| *CYP3A4* | Cytochrome P450, family 3, subfamily A, polypeptide 4 | -1.497 | 4.5E-05 |
| *CYP4B1* | Cytochrome P450, family 4, subfamily B, polypeptide 1 | -4.366 | 5.02E-05 |
| *CYSLTR2* | Cysteinyl leukotriene receptor 2 | 1.448 | 0.0055 |
| *DAAM1* | Cishevelled associated activator of morphogenesis 1 | -1.543 | 2.91E-08 |
| *DAAM2* | Cishevelled associated activator of morphogenesis 2 | -1.749 | 4.52E-08 |
| *DAB2IP* | DAB2 interacting protein | -1.324 | 0.00421 |
| *DAO* | D-amino-acid oxidase | -1.534 | 1.09E-05 |
| *DAPK2* | Death-associated protein kinase 2 | 2.112 | 1.35E-05 |
| *DCAF11* | DDB1 and CUL4 associated factor 11 | -1.268 | 0.00239 |
| *DCPS* | Decapping enzyme, scavenger | 1.385 | 0.00099 |
| *DCTN4* | Dynactin 4 (p62) | -1.261 | 0.00172 |
| *DCXR* | Dicarbonyl/L-xylulose reductase | -1.319 | 0.00126 |
| *DDAH1* | Dimethylarginine dimethylaminohydrolase 1 | -1.438 | 0.00025 |
| *DDC* | Dopa decarboxylase (aromatic L-amino acid decarboxylase) | -1.644 | 1.63E-06 |
| *DDIT3* | DNA-damage-inducible transcript 3 | 1.681 | 5.63E-06 |
| *DDT* | D-dopachrome tautomerase | 1.535 | 2.42E-05 |
| *DDX3X* | DEAD (Asp-Glu-Ala-Asp) box helicase 3, X-linked | -1.281 | 0.0015 |
| *DECR1* | 2,4-dienoyl CoA reductase 1, mitochondrial | -1.291 | 0.00271 |
| *DEGS1* | Delta(4)-desaturase, sphingolipid 1 | -1.404 | 4.68E-06 |
| *DENND2A* | DENN/MADD domain containing 2A | 2.68 | 1.59E-11 |
| *DENND5B* | DENN/MADD domain containing 5B | -1.477 | 0.0012 |
| *DERL3* | Derlin 3 | 2.778 | 8.96E-08 |
| *DES* | Desmin | 1.361 | 0.0047 |
| *DFNB31* | Deafness, autosomal recessive 31 | -2.045 | 2.39E-09 |
| *DGAT2* | Diacylglycerol O-acyltransferase 2 | 1.559 | 3.09E-09 |
| *DHCR24* | 24-dehydrocholesterol reductase | -1.941 | 3.40E-09 |
| *DHFR* | Dihydrofolate reductase | -1.294 | 0.00352 |
| *DHTKD1* | Dehydrogenase E1 and transketolase domain containing 1 | -1.35 | 0.00057 |
| *DIO1* | Deiodinase, iodothyronine, type I | -1.719 | 8.98E-05 |
| *DIP2A* | DIP2 disco-interacting protein 2 homolog A (Drosophila) | -1.365 | 0.0003 |
| *DIRC2* | Disrupted in renal carcinoma 2 | -1.296 | 0.00343 |
| *DKK3* | Dickkopf WNT signaling pathway inhibitor 3 | -1.776 | 6.24E-05 |
| *DKKL1* | Dickkopf-like 1 | 1.39 | 0.00148 |
| *DLAT* | Dihydrolipoamide S-acetyltransferase | -1.407 | 3.21E-05 |
| *DLC1* | Deleted in liver cancer 1 | 1.347 | 0.00487 |
| *DLG1* | Discs, large homolog 1 (Drosophila) | -1.276 | 0.00569 |
| *DLST* | Dihydrolipoamide S-succinyltransferase (E2 component of 2-oxo-glutarate complex) | -1.366 | 0.00048 |
| *DNAJB1* | DnaJ (Hsp40) homolog, subfamily B, member 1 | 1.338 | 0.00301 |
| *DNAJB11* | DnaJ (Hsp40) homolog, subfamily B, member 11 | 1.415 | 0.00481 |
| *DNAJC12* | DnaJ (Hsp40) homolog, subfamily C, member 12 | 1.533 | 2.87E-06 |
| *DNAJC18* | DnaJ (Hsp40) homolog, subfamily C, member 18 | 1.399 | 0.00034 |
| *DNAJC22* | DnaJ (Hsp40) homolog, subfamily C, member 22 | -1.397 | 0.00016 |
| *DNPH1* | 2'-deoxynucleoside 5'-phosphate N-hydrolase 1 | 1.426 | 0.00072 |
| *DOK4* | Docking protein 4 | -1.461 | 1.21E-05 |
| *DPM3* | Dolichyl-phosphate mannosyltransferase polypeptide 3 | 1.577 | 0.00052 |
| *DPY19L3* | Dpy-19-like 3 (C. elegans) | -1.609 | 0.00088 |
| *DPYD* | Dihydropyrimidine dehydrogenase | -1.341 | 0.0006 |
| *DRAM2* | DNA-damage regulated autophagy modulator 2 | -1.342 | 0.00051 |
| *DST* | Dystonin | -1.498 | 1.34E-05 |
| *DUSP26* | Dual specificity phosphatase 26 (putative) | 2.523 | 0.00084 |
| *DYNC2LI1* | Dynein, cytoplasmic 2, light intermediate chain 1 | 1.631 | 0.00535 |
| *DYNLL1* | Dynein, light chain, LC8-type 1 | 1.346 | 0.00626 |
| *DYRK1A* | Dual-specificity tyrosine-(Y)-phosphorylation regulated kinase 1A | -1.262 | 0.00276 |
| *DYRK1B* | Dual-specificity tyrosine-(Y)-phosphorylation regulated kinase 1B | -1.36 | 0.0012 |
| *DZIP3* | DAZ interacting zinc finger protein 3 | -1.802 | 0.00103 |
| *EBF1* | Early B-cell factor 1 | -1.662 | 0.00089 |
| *ECE1* | Endothelin converting enzyme 1 | -1.447 | 6.37E-06 |
| *ECM1* | Extracellular matrix protein 1 | 1.582 | 0.00012 |
| *EDEM1* | ER degradation enhancer, mannosidase alpha-like 1 | -1.414 | 0.00534 |
| *EEF1B2* | Eukaryotic translation elongation factor 1 beta 2 | 1.281 | 0.00198 |
| *EEF2K* | Eukaryotic elongation factor-2 kinase | -1.479 | 1.88E-06 |
| *EFNA4* | Ephrin-A4 | 1.251 | 0.00407 |
| *EFNB1* | Ephrin-B1 | 1.359 | 0.0159 |
| *EGFR* | Epidermal growth factor receptor | -1.428 | 0.0105 |
| *EHHADH* | Enoyl-CoA, hydratase/3-hydroxyacyl CoA dehydrogenase | -1.483 | 0.00025 |
| *EIF4EBP1* | Eukaryotic translation initiation factor 4E binding protein 1 | 1.412 | 1.37E-05 |
| *EIF4EBP2* | Eukaryotic translation initiation factor 4E binding protein 2 | -1.425 | 0.00236 |
| *EIF5* | Eukaryotic translation initiation factor 5 | -1.315 | 0.00152 |
| *ELK1* | ELK1, member of ETS oncogene family | -1.329 | 0.00067 |
| *ELOVL6* | ELOVL fatty acid elongase 6 | -1.879 | 8.77E-08 |
| *EML2* | Echinoderm microtubule associated protein like 2 | 1.503 | 0.00043 |
| *ENO3* | Enolase 3 (beta, muscle) | 1.573 | 8.05E-05 |
| *ENO4* | Enolase family member 4 | 1.689 | 0.00015 |
| *ENPEP* | Glutamyl aminopeptidase (aminopeptidase A) | -1.354 | 0.00035 |
| *ENPP1* | Ectonucleotide pyrophosphatase/phosphodiesterase 1 | -1.54 | 3.05E-05 |
| *EPS15* | Epidermal growth factor receptor pathway substrate 15 | -1.271 | 0.00066 |
| *ERCC6L2* | Excision repair cross-complementation group 6-like 2 | -1.335 | 2.68E-05 |
| *ERO1B* | Endoplasmic reticulum oxidoreductase beta | -1.848 | 1.82E-08 |
| *EXOSC4* | Exosome component 4 | 1.369 | 0.0012 |
| *EXTL1* | Exostosin-like glycosyltransferase 1 | 1.885 | 1.58E-06 |
| *EXTL3* | Exostosin-like glycosyltransferase 3 | -1.403 | 2.51E-05 |
| *Fam13a* | Family with sequence similarity 13, member A | -1.953 | 2.49E-06 |
| *FAM167B* | Family with sequence similarity 167, member B | 1.9 | 6.58E-05 |
| *FAM188A* | Family with sequence similarity 188, member A | 1.273 | 0.0007 |
| *FAM189A2* | Family with sequence similarity 189, member A2 | -1.427 | 0.00423 |
| *FAM20B* | Family with sequence similarity 20, member B | -1.401 | 0.00292 |
| *FAM210B* | Family with sequence similarity 210, member B | 1.817 | 1.70E-07 |
| *FAM213B* | Family with sequence similarity 213, member B | 1.509 | 1.40E-06 |
| *FAM35A* | Family with sequence similarity 35, member A | 1.285 | 0.004 |
| *FAM47E* | Family with sequence similarity 47, member E | 2.143 | 0.0003 |
| *FAM58A* | Family with sequence similarity 58, member A | 1.368 | 6.05E-05 |
| *FAM69A* | Family with sequence similarity 69, member A | -1.716 | 7.39E-08 |
| *FAM91A1* | Family with sequence similarity 91, member A1 | -1.404 | 2.9E-05 |
| *FAU* | Finkel-Biskis-Reilly murine sarcoma virus (FBR-MuSV) ubiquitously expressed | 1.553 | 1.38E-05 |
| *FBXL14* | F-box and leucine-rich repeat protein 14 | 1.478 | 5.02E-05 |
| *FBXO17* | F-box protein 17 | 1.417 | 0.00146 |
| *FBXO42* | F-box protein 42 | -1.412 | 4.82E-05 |
| *FBXO44* | F-box protein 44 | -1.861 | 2.51E-09 |
| *FDFT1* | Farnesyl-diphosphate farnesyltransferase 1 | 1.682 | 0.00095 |
| *FECH* | Ferrochelatase | -1.585 | 1.27E-11 |
| *FETUB* | Fetuin B | 2.875 | 5.43E-09 |
| *FGD1* | FYVE, RhoGEF and PH domain containing 1 | 1.447 | 0.00389 |
| *FGFR4* | Fibroblast growth factor receptor 4 | -1.349 | 0.00041 |
| *FGGY* | FGGY carbohydrate kinase domain containing | -1.302 | 0.00179 |
| *FITM2* | Fat storage-inducing transmembrane protein 2 | -1.519 | 8.67E-06 |
| *FKBP11* | FK506 binding protein 11, 19 kDa | 1.438 | 0.00039 |
| *FLRT2* | Fibronectin leucine rich transmembrane protein 2 | -2.387 | 0.00011 |
| *FLT3LG* | Fms-related tyrosine kinase 3 ligand | 1.821 | 3.20E-08 |
| *FMO1* | Flavin containing monooxygenase 1 | 1.659 | 3.99E-06 |
| *FMO2* | Flavin containing monooxygenase 2 (non-functional) | 1.833 | 0.00031 |
| *FNDC4* | Fibronectin type III domain containing 4 | 2.115 | 3.07E-06 |
| *FNIP1* | Folliculin interacting protein 1 | -1.359 | 0.00488 |
| *FOSL2* | FOS-like antigen 2 | 1.457 | 0.00034 |
| *FOXO3* | Fforkhead box O3 | -1.799 | 4.33E-05 |
| *FOXO4* | Forkhead box O4 | 1.359 | 9.55E-05 |
| *FOXP1* | Forkhead box P1 | 1.469 | 0.00235 |
| *FSD2* | Fibronectin type III and SPRY domain containing 2 | -1.607 | 0.00276 |
| *FST* | Follistatin | 2.878 | 7.13E-10 |
| *FTL* | Fferritin, light polypeptide | 1.633 | 8.86E-06 |
| *FUOM* | Fucose mutarotase | 2.128 | 0.00017 |
| *FYN* | FYN oncogene related to SRC, FGR, YES | 1.341 | 0.00118 |
| *FZD4* | Frizzled family receptor 4 | -1.406 | 0.00251 |
| *G0S2* | G0/G1switch 2 | 3.376 | 0.00029 |
| *GADD45GIP1* | Growth arrest and DNA-damage-inducible, gamma interacting protein 1 | 1.446 | 0.00019 |
| *GALE* | UDP-galactose-4-epimerase | -1.46 | 0.00192 |
| *GALK1* | Galactokinase 1 | 1.873 | 7.60E-08 |
| *GALNT1* | UDP-N-acetyl-alpha-D-galactosamine:polypeptide N-acetylgalactosaminyltransferase 1 (GalNAc-T1) | -1.292 | 0.00075 |
| *GALNT10* | Polypeptide N-acetylgalactosaminyltransferase 10 | -2.066 | 9.03E-07 |
| *GALNT14* | UDP-N-acetyl-alpha-D-galactosamine:polypeptide N-acetylgalactosaminyltransferase 14 (GalNAc-T14) | -2.757 | 1.32E-06 |
| *GARS* | Glycyl-tRNA synthetase | 1.501 | 1.45E-06 |
| *GAS6* | Growth arrest-specific 6 | 1.487 | 0.0003 |
| *GAS8* | Growth arrest-specific 8 | 1.418 | 1.07E-05 |
| *GATA4* | GATA binding protein 4 | 1.329 | 0.00549 |
| *GBGT1* | Globoside alpha-1,3-N-acetylgalactosaminyltransferase 1 | 1.454 | 0.00443 |
| *GCC2* | GRIP and coiled-coil domain containing 2 | -1.326 | 0.00047 |
| *GDA* | Guanine deaminase | -1.262 | 0.00163 |
| *GDPD5* | Glycerophosphodiester phosphodiesterase domain containing 5 | -1.723 | 7.09E-05 |
| *GFM2* | G elongation factor, mitochondrial 2 | -1.321 | 0.00264 |
| *GGT1* | Gamma-glutamyltransferase 1 | -1.382 | 0.00043 |
| *GHR* | Growth hormone receptor | -2.013 | 5.03E-10 |
| *GIMAP1* | GTPase, IMAP family member 1 | 1.887 | 0.00215 |
| *GIMAP1-GIMAP5* | GIMAP1-GIMAP5 readthrough | 1.944 | 0.00033 |
| *GIMAP4* | GTPase, IMAP family member 4 | 1.571 | 0.00167 |
| *GK5* | Glycerol kinase 5 (putative) | -1.722 | 1.61E-07 |
| *GLDC* | Glycine dehydrogenase (decarboxylating) | -2.191 | 3.91E-11 |
| *GLI1* | GLI family zinc finger 1 | 2.054 | 0.00291 |
| *GLS2* | Glutaminase 2 (liver, mitochondrial) | -2.024 | 1.19E-07 |
| *GLTPD2* | Glycolipid transfer protein domain containing 2 | -1.824 | 6.20E-07 |
| *GLTSCR2* | Glioma tumor suppressor candidate region gene 2 | 1.408 | 0.00013 |
| *GNAQ* | Guanine nucleotide binding protein (G protein), q polypeptide | -1.413 | 6.77E-05 |
| *GNB2L1* | Guanine nucleotide binding protein (G protein), beta polypeptide 2-like 1 | 1.276 | 0.00418 |
| *GNG5* | Guanine nucleotide binding protein (G protein), gamma 5 | 1.319 | 0.00035 |
| *GOLGA7* | Golgin A7 | 1.311 | 4.62E-05 |
| *GOLGB1* | Golgin B1 | -1.288 | 0.00483 |
| *GOLIM4* | Golgi integral membrane protein 4 | -1.459 | 7.49E-05 |
| *GOLT1A* | Golgi transport 1A | 1.527 | 3.63E-07 |
| *GOT1* | Glutamic-oxaloacetic transaminase 1, soluble | -1.494 | 0.00012 |
| *GPC3* | Glypican 3 | 4.752 | 1.61E-10 |
| *GPC4* | Glypican 4 | -1.849 | 0.00141 |
| *GPHN* | Gephyrin | -1.425 | 3.16E-05 |
| *GPLD1* | Glycosylphosphatidylinositol specific phospholipase D1 | -1.647 | 6.75E-07 |
| *GPR107* | G protein-coupled receptor 107 | -1.54 | 0.00068 |
| *GPR182* | G protein-coupled receptor 182 | 1.335 | 0.00454 |
| *GPRC5A* | G protein-coupled receptor, family C, group 5, member A | -1.59 | 4.27E-05 |
| *GPX3* | Glutathione peroxidase 3 (plasma) | 8.481 | 6.70E-11 |
| *GRASP* | GRP1 (general receptor for phosphoinositides 1)-associated scaffold protein | 1.644 | 0.00138 |
| *GSDMB* | Gasdermin B | -2.533 | 1.32E-13 |
| *GSS* | Glutathione synthetase | -1.312 | 0.00133 |
| *GSTA4* | Glutathione S-transferase alpha 4 | -1.724 | 0.001 |
| *GSTM1* | Glutathione S-transferase mu 1 | -1.566 | 0.00087 |
| *GSTM3* | Glutathione S-transferase mu 3 (brain) | -1.448 | 0.00041 |
| *GSTM4* | Glutathione S-transferase mu 4 | -3.963 | 5.11E-20 |
| *GTF3C4* | General transcription factor IIIC, polypeptide 4, 90kDa | -1.437 | 0.00484 |
| *GTPBP2* | GTP binding protein 2 | 1.394 | 0.00116 |
| *Gulo* | Gulonolactone (L-) oxidase | 1.688 | 3.70E-07 |
| *HACL1* | 2-hydroxyacyl-CoA lyase 1 | -1.489 | 1.25E-05 |
| *HADH* | Hydroxyacyl-CoA dehydrogenase | -1.294 | 0.0021 |
| *HADHB* | Hydroxyacyl-CoA dehydrogenase/3-ketoacyl-CoA thiolase/enoyl-CoA hydratase (trifunctional protein), beta subunit | 1.3 | 0.00525 |
| *HAUS3* | HAUS augmin-like complex, subunit 3 | 1.357 | 0.0041 |
| *HBB* | Hemoglobin, beta | -3.409 | 0.00086 |
| *HDC* | Histidine decarboxylase | 1.751 | 0.00156 |
| *HERC2* | HECT and RLD domain containing E3 ubiquitin protein ligase 2 | -1.287 | 0.00121 |
| *HERPUD1* | Homocysteine-inducible, endoplasmic reticulum stress-inducible, ubiquitin-like domain member 1 | 1.625 | 9.3E-05 |
| *HEY1* | Hairy/enhancer-of-split related with YRPW motif 1 | -1.753 | 0.00274 |
| *HIST1H2BD* | Histone cluster 1, H2bd | -2.6 | 5.98E-12 |
| *HIST1H2BE* | Histone cluster 1, H2be | -1.496 | 0.0058 |
| *HIST1H2BO* | Histone cluster 1, H2bo | -1.357 | 0.00192 |
| *HIST1H4J* | Histone cluster 1, H4j | 1.639 | 0.00091 |
| *HIST2H4A* | Histone cluster 2, H4a | -2.775 | 5.79E-10 |
| *HMGCS2* | 3-hydroxy-3-methylglutaryl-CoA synthase 2 (mitochondrial) | -1.479 | 0.0043 |
| *HMHA1* | Histocompatibility (minor) HA-1 | 1.451 | 0.00227 |
| *HNRNPA1* | Heterogeneous nuclear ribonucleoprotein A1 | 1.334 | 0.00031 |
| *HOGA1* | 4-hydroxy-2-oxoglutarate aldolase 1 | -1.829 | 2.23E-08 |
| *HOMER2* | Homer homolog 2 (Drosophila) | 1.431 | 4.90E-06 |
| *HOOK1* | Hook microtubule-tethering protein 1 | -1.486 | 0.0014 |
| *HPCAL4* | Hippocalcin like 4 | -2.189 | 0.00274 |
| *HPGD* | Hydroxyprostaglandin dehydrogenase 15-(NAD) | -1.387 | 0.00514 |
| *HPX* | Hemopexin | -1.747 | 0.00093 |
| *HSD17B12* | Hhydroxysteroid (17-beta) dehydrogenase 12 | -1.456 | 0.00392 |
| *HSD17B13* | Hydroxysteroid (17-beta) dehydrogenase 13 | -1.579 | 0.00131 |
| *HSD17B6* | Hydroxysteroid (17-beta) dehydrogenase 6 | -1.506 | 8.98E-05 |
| *HSDL2* | Hydroxysteroid dehydrogenase like 2 | 1.519 | 2.64E-07 |
| *HSF4* | Heat shock transcription factor 4 | 1.89 | 0.00475 |
| *HSPA6* | Heat shock 70kDa protein 6 (HSP70B') | 3.86 | 1.94E-07 |
| *HSPB1* | Heat shock 27kDa protein 1 | 1.514 | 0.00044 |
| *HTATIP2* | HIV-1 Tat interactive protein 2, 30kDa | 1.529 | 1.43E-05 |
| *HTR2B* | 5-hydroxytryptamine (serotonin) receptor 2B, G protein-coupled | -2.111 | 3.30E-07 |
| *ID2* | Inhibitor of DNA binding 2, dominant negative helix-loop-helix protein | 1.487 | 0.00046 |
| *IDE* | Insulin-degrading enzyme | -1.321 | 0.00523 |
| *IDH1* | Isocitrate dehydrogenase 1 (NADP+), soluble | 1.337 | 0.00022 |
| *IDH2* | Isocitrate dehydrogenase 2 (NADP+), mitochondrial | 1.604 | 2.77E-10 |
| *IER3* | Immediate early response 3 | 1.402 | 0.00365 |
| *IFITM1* | Interferon induced transmembrane protein 1 | 1.673 | 2.31E-05 |
| *IFRD1* | Interferon-related developmental regulator 1 | 1.536 | 0.00124 |
| *Ift20* | Intraflagellar transport 20 | 1.311 | 0.00294 |
| *IFT27* | Intraflagellar transport 27 homolog (Chlamydomonas) | 1.673 | 0.00145 |
| *IGDCC4* | Immunoglobulin superfamily, DCC subclass, member 4 | 5.901 | 1.61E-16 |
| *IGF1* | Insulin-like growth factor 1 (somatomedin C) | -2.701 | 1.93E-11 |
| *IGF2R* | Insulin-like growth factor 2 receptor | -1.297 | 0.0048 |
| *IGFBP1* | Insulin-like growth factor binding protein 1 | 3.411 | 2.68E-05 |
| *IGFBP2* | Insulin-like growth factor binding protein 2, 36kDa | 27.595 | 1.38E-17 |
| *IGFBP6* | Insulin-like growth factor binding protein 6 | 1.729 | 9.2E-05 |
| *IGSF11* | Immunoglobulin superfamily, member 11 | -1.463 | 0.00029 |
| *IGSF5* | Immunoglobulin superfamily, member 5 | -1.392 | 4.59E-05 |
| *IHH* | Indian hedgehog | 1.906 | 4.7E-05 |
| *IKBKAP* | Inhibitor of kappa light polypeptide gene enhancer in B-cells, kinase complex-associated protein | -1.345 | 0.00379 |
| *IKBKB* | Inhibitor of kappa light polypeptide gene enhancer in B-cells, kinase beta | -1.297 | 0.00047 |
| *IL17RB* | Interleukin 17 receptor B | -2.069 | 0.00248 |
| *IL17RC* | Interleukin 17 receptor C | 1.275 | 0.00484 |
| *IL1R2* | Interleukin 1 receptor, type II | -3.171 | 1.78E-05 |
| *INHBA* | Inhibin, beta A | 3.007 | 2.72E-07 |
| *INHBC* | Iinhibin, beta C | -1.759 | 0.00027 |
| *INHBE* | Inhibin, beta E | 1.585 | 0.00379 |
| *INMT* | Indolethylamine N-methyltransferase | 2.2 | 0.00066 |
| *INPP5A* | Inositol polyphosphate-5-phosphatase, 40kDa | -1.266 | 0.00424 |
| *INPP5F* | Inositol polyphosphate-5-phosphatase F | -1.355 | 0.00063 |
| *INSIG1* | Insulin induced gene 1 | 1.715 | 7.9E-05 |
| *IREB2* | Iron-responsive element binding protein 2 | -1.364 | 0.00045 |
| *IRS2* | Insulin receptor substrate 2 | 1.818 | 0.00084 |
| *ITGA7* | Integrin, alpha 7 | -1.394 | 0.00169 |
| *ITGAV* | Integrin, alpha V | -1.378 | 0.00482 |
| *ITPR1* | Inositol 1,4,5-trisphosphate receptor, type 1 | -1.705 | 2.67E-08 |
| *ITPR2* | Inositol 1,4,5-trisphosphate receptor, type 2 | -1.369 | 0.00306 |
| *ITSN2* | Intersectin 2 | -1.33 | 5.17E-05 |
| *IVD* | Isovaleryl-CoA dehydrogenase | -1.625 | 3.69E-09 |
| *IYD* | Iiodotyrosine deiodinase | -1.351 | 0.00087 |
| *JAG1* | Jagged 1 | 1.325 | 0.00169 |
| *JAK3* | Janus kinase 3 | 1.524 | 0.0027 |
| *JAKMIP2* | Janus kinase and microtubule interacting protein 2 | -1.91 | 0.00205 |
| *JCHAIN* | Joining chain of multimeric IgA and IgM | 2.004 | 0.00283 |
| *JMJD6* | Jumonji domain containing 6 | 1.495 | 0.00061 |
| *KBTBD6* | Kelch repeat and BTB (POZ) domain containing 6 | 1.827 | 0.00011 |
| *KCNH3* | Potassium voltage-gated channel, subfamily H (eag-related), member 3 | 2.077 | 1.69E-07 |
| *KCNJ12* | Potassium inwardly-rectifying channel, subfamily J, member 12 | -2.247 | 7.20E-09 |
| *KCNMA1* | Potassium large conductance calcium-activated channel, subfamily M, alpha member 1 | -1.466 | 0.00364 |
| *KCTD3* | Potassium channel tetramerization domain containing 3 | -1.431 | 0.00023 |
| *KDM4B* | Lysine (K)-specific demethylase 4B | -1.34 | 9.53E-05 |
| *KIAA0825* | KIAA0825 | -1.899 | 1.39E-05 |
| *KIAA1217* | KIAA1217 | -1.296 | 0.00207 |
| *KIAA1467* | KIAA1467 | -1.467 | 4.31E-06 |
| *KIAA1522* | KIAA1522 | -1.422 | 0.00098 |
| *KIAA1715* | KIAA1715 | -1.314 | 0.00386 |
| *KIF1B* | Kinesin family member 1B | -1.581 | 1.33E-05 |
| *KIF20A* | Kinesin family member 20A | -2.291 | 0.00098 |
| *KIF21A* | Kinesin family member 21A | -1.464 | 0.00018 |
| *KITLG* | KIT ligand | -1.855 | 0.00307 |
| *KLF9* | Kruppel-like factor 9 | -1.545 | 9.69E-06 |
| *KLHL13* | Kelch-like family member 13 | -1.652 | 8.58E-07 |
| *KLHL24* | Kelch-like family member 24 | -1.575 | 0.00019 |
| *KLKB1* | Kallikrein B, plasma (Fletcher factor) 1 | -1.388 | 0.00116 |
| *Klre1* | Killer cell lectin-like receptor family E member 1 | 1.856 | 0.00472 |
| *KMO* | Kynurenine 3-monooxygenase (kynurenine 3-hydroxylase) | -2.469 | 5.62E-17 |
| *LARGE* | Like-glycosyltransferase | -1.38 | 1.59E-05 |
| *LAT* | Linker for activation of T cells | 1.703 | 0.00158 |
| *LCK* | Lymphocyte-specific protein tyrosine kinase | 1.794 | 5.46E-05 |
| *LDAH* | Lipid droplet associated hydrolase | -1.606 | 8.11E-06 |
| *LEPR* | Leptin receptor | 1.682 | 9.67E-05 |
| *LGALS8* | Lectin, galactoside-binding, soluble, 8 | -1.598 | 4.85E-05 |
| *LGMN* | Legumain | -1.594 | 1.93E-06 |
| *LGR4* | Leucine-rich repeat containing G protein-coupled receptor 4 | -1.938 | 1.22E-08 |
| *LHX2* | LIM homeobox 2 | 1.643 | 0.00104 |
| *LIMA1* | LIM domain and actin binding 1 | -1.371 | 0.00059 |
| *LIMS2* | LIM and senescent cell antigen-like domains 2 | -1.399 | 0.00272 |
| *LIN7B* | Lin-7 homolog B (C. elegans) | 1.901 | 4.65E-05 |
| *LLGL2* | Lethal giant larvae homolog 2 (Drosophila) | -1.315 | 0.00021 |
| *LLPH* | LLP homolog, long-term synaptic facilitation (Aplysia) | 1.275 | 0.00377 |
| *LMAN2L* | Lectin, mannose-binding 2-like | -1.308 | 0.00084 |
| *LMTK2* | Lemur tyrosine kinase 2 | -1.39 | 0.00136 |
| *LOXL4* | Lysyl oxidase-like 4 | 2.568 | 5.80E-06 |
| *LPCAT3* | Lysophosphatidylcholine acyltransferase 3 | 1.404 | 0.00108 |
| *LPIN1* | Lipin 1 | -1.538 | 0.00581 |
| *LPIN2* | Lipin 2 | -1.41 | 0.00062 |
| *LRBA* | LPS-responsive vesicle trafficking, beach and anchor containing | -1.318 | 0.00155 |
| *LRIG3* | Leucine-rich repeats and immunoglobulin-like domains 3 | -1.886 | 0.00228 |
| *LRP6* | Low density lipoprotein receptor-related protein 6 | -1.608 | 0.00013 |
| *LSM3* | LSM3 homolog, U6 small nuclear RNA associated (S. cerevisiae) | 1.317 | 0.00062 |
| *LY6E* | Lymphocyte antigen 6 complex, locus E | -1.889 | 8.52E-06 |
| *LZTS2* | Leucine zipper, putative tumor suppressor 2 | 1.372 | 0.00361 |
| *MAGED1* | Melanoma antigen family D, 1 | 1.319 | 0.00084 |
| *MAGEH1* | Melanoma antigen family H, 1 | 1.435 | 1.04E-05 |
| *MAGI1* | Membrane associated guanylate kinase, WW and PDZ domain containing 1 | -1.43 | 0.00018 |
| *MAMDC2* | MAM domain containing 2 | -2.597 | 4.66E-05 |
| *MAN2B1* | Mannosidase, alpha, class 2B, member 1 | -1.289 | 0.00243 |
| *MANF* | Mesencephalic astrocyte-derived neurotrophic factor | 1.659 | 0.00072 |
| *MAOB* | Monoamine oxidase B | -1.495 | 3.38E-05 |
| *MAP1LC3A* | Microtubule-associated protein 1 light chain 3 alpha | 1.418 | 0.00046 |
| *MAP2* | Microtubule-associated protein 2 | -1.441 | 0.00488 |
| *MAP2K6* | Mitogen-activated protein kinase kinase 6 | -1.652 | 0.00026 |
| *MAPK6* | Mitogen-activated protein kinase 6 | -2.001 | 2.38E-07 |
| *MAPK9* | Mitogen-activated protein kinase 9 | -1.425 | 0.00061 |
| *MAPRE3* | Microtubule-associated protein, RP/EB family, member 3 | 1.329 | 9.09E-05 |
| *MARS* | Methionyl-tRNA synthetase | 1.477 | 7.82E-05 |
| *MAT2A* | Methionine adenosyltransferase II, alpha | 1.886 | 3.35E-15 |
| *MBP* | Myelin basic protein | -1.536 | 0.00283 |
| *MCCC2* | Methylcrotonoyl-CoA carboxylase 2 (beta) | -1.333 | 0.00209 |
| *MDM1* | Mdm1 nuclear protein homolog (mouse) | 1.484 | 0.00526 |
| *ME1* | Malic enzyme 1, NADP(+)-dependent, cytosolic | 3.108 | 6.78E-07 |
| *MED31* | Mediator complex subunit 31 | 1.279 | 0.00152 |
| *MEP1B* | Meprin A, beta | -2.363 | 1.56E-05 |
| *METTL1* | Methyltransferase like 1 | 1.411 | 0.00163 |
| *MIB1* | Mindbomb E3 ubiquitin protein ligase 1 | -1.405 | 0.0008 |
| *MIER3* | Mesoderm induction early response 1, family member 3 | -1.442 | 0.00127 |
| *MIF* | Macrophage migration inhibitory factor (glycosylation-inhibiting factor) | 1.854 | 3.08E-05 |
| *MIF4GD* | MIF4G domain containing | -1.425 | 4.02E-05 |
| *MIOX* | Myo-inositol oxygenase | 17.779 | 7.40E-26 |
| *MKL2* | MKL/myocardin-like 2 | -1.374 | 0.00105 |
| *MKNK2* | MAP kinase interacting serine/threonine kinase 2 | -1.366 | 0.00062 |
| *MLEC* | Malectin | -1.546 | 5.96E-07 |
| *MLF2* | Myeloid leukemia factor 2 | -1.332 | 0.00058 |
| *MMP11* | Matrix metallopeptidase 11 (stromelysin 3) | -1.652 | 0.00029 |
| *MMP15* | Matrix metallopeptidase 15 (membrane-inserted) | -1.798 | 1.24E-07 |
| *MMP23B* | Matrix metallopeptidase 23B | -3.608 | 7.18E-08 |
| *MOGAT1* | Monoacylglycerol O-acyltransferase 1 | -2.007 | 4.42E-08 |
| *MOXD1* | Monooxygenase, DBH-like 1 | -1.77 | 0.00462 |
| *MPC1* | Mitochondrial pyruvate carrier 1 | 1.537 | 9.29E-05 |
| *MPP5* | Membrane protein, palmitoylated 5 (MAGUK p55 subfamily member 5) | -1.459 | 1.76E-05 |
| *MRPL11* | Mitochondrial ribosomal protein L11 | 1.34 | 0.00023 |
| *MRPL15* | Mitochondrial ribosomal protein L15 | 1.522 | 3.74E-06 |
| *MRPL20* | Mitochondrial ribosomal protein L20 | 1.269 | 0.00291 |
| *MRPL40* | Mitochondrial ribosomal protein L40 | 1.364 | 0.00097 |
| *MRPL41* | Mitochondrial ribosomal protein L41 | 1.34 | 0.00426 |
| *MRPL52* | Mitochondrial ribosomal protein L52 | 1.312 | 0.00037 |
| *MRPS14* | Mitochondrial ribosomal protein S14 | 1.281 | 0.00172 |
| *MRPS16* | Mitochondrial ribosomal protein S16 | 1.367 | 6.32E-05 |
| *MRPS17* | Mitochondrial ribosomal protein S17 | 1.335 | 0.00054 |
| *MRPS18A* | Mitochondrial ribosomal protein S18A | 1.329 | 0.0027 |
| *MRPS18C* | Mitochondrial ribosomal protein S18C | 1.323 | 0.00251 |
| *MSTO1* | Misato 1, mitochondrial distribution and morphology regulator | 1.361 | 0.00349 |
| *MTHFR* | Methylenetetrahydrofolate reductase (NAD(P)H) | -1.697 | 0.00151 |
| *MVD* | Mevalonate (diphospho) decarboxylase | 1.517 | 0.00153 |
| *MYEF2* | Myelin expression factor 2 | -1.988 | 0.00021 |
| *MYH14* | Myosin, heavy chain 14, non-muscle | -1.572 | 1.56E-06 |
| *MYLK4* | Myosin light chain kinase family, member 4 | -2.048 | 0.00211 |
| *MYO1D* | Myosin ID | -1.323 | 0.00146 |
| *MYO5B* | Myosin VB | -1.558 | 6.41E-09 |
| *MZB1* | Marginal zone B and B1 cell-specific protein | 3.047 | 0.00159 |
| *MZT2B* | Mitotic spindle organizing protein 2B | 1.51 | 0.0014 |
| *N4BP2* | NEDD4 binding protein 2 | -1.428 | 0.0019 |
| *NADK* | NAD kinase | -1.319 | 0.00097 |
| *NATD1* | N-acetyltransferase domain containing 1 | -1.439 | 0.00332 |
| *NAV3* | Neuron navigator 3 | -1.602 | 0.00015 |
| *NBAS* | Neuroblastoma amplified sequence | -1.286 | 0.00176 |
| *NCKAP5* | NCK-associated protein 5 | -1.658 | 0.00012 |
| *NCOA2* | Nnuclear receptor coactivator 2 | -1.356 | 0.00325 |
| *NCOR1* | Nuclear receptor corepressor 1 | -1.259 | 0.00095 |
| *NCOR2* | Nuclear receptor corepressor 2 | -1.432 | 6.96E-05 |
| *NDUFAF2* | NADH dehydrogenase (ubiquinone) complex I, assembly factor 2 | 1.49 | 0.0157 |
| *NDUFB10* | NADH dehydrogenase (ubiquinone) 1 beta subcomplex, 10, 22kDa | 1.425 | 3.38E-06 |
| *NDUFC2* | NADH dehydrogenase (ubiquinone) 1, subcomplex unknown, 2, 14.5kDa | 1.285 | 0.0006 |
| *NEBL* | Nebulette | -1.542 | 0.00443 |
| *NEDD9* | Neural precursor cell expressed, developmentally down-regulated 9 | 1.345 | 0.00146 |
| *NEK9* | NIMA-related kinase 9 | -1.329 | 0.00019 |
| *NELFE* | Negative elongation factor complex member E | 1.269 | 0.00185 |
| *NETO2* | Neuropilin (NRP) and tolloid (TLL)-like 2 | -1.911 | 1.75E-06 |
| *NFIA* | Nuclear factor I/A | -1.377 | 0.00238 |
| *NFKBIZ* | Nuclear factor of kappa light polypeptide gene enhancer in B-cells inhibitor, zeta | 1.569 | 3.54E-06 |
| *NFYB* | Nuclear transcription factor Y, beta | -1.382 | 0.00141 |
| *NHLRC1* | NHL repeat containing 1 | 1.687 | 0.00101 |
| *NHLRC3* | NHL repeat containing 3 | -1.348 | 0.00148 |
| *NHSL1* | NHS-like 1 | -1.534 | 0.00019 |
| *NID1* | Nidogen 1 | -1.459 | 0.00012 |
| *NKD1* | Naked cuticle homolog 1 (Drosophila) | 2.117 | 0.00053 |
| *NLRC5* | NLR family, CARD domain containing 5 | 1.613 | 0.00084 |
| *NLRX1* | NLR family member X1 | -1.332 | 0.00122 |
| *NME3* | NME/NM23 nucleoside diphosphate kinase 3 | 1.443 | 0.00062 |
| *NME6* | NME/NM23 nucleoside diphosphate kinase 6 | 1.367 | 0.00216 |
| *NNAT* | Neuronatin | 7.565 | 3.32E-16 |
| *NOD2* | Nucleotide-binding oligomerization domain containing 2 | -1.655 | 0.00098 |
| *NOSIP* | Nitric oxide synthase interacting protein | 1.267 | 0.00557 |
| *NR0B2* | Nuclear receptor subfamily 0, group B, member 2 | -2.115 | 3.18E-05 |
| *NR1D1* | Nuclear receptor subfamily 1, group D, member 1 | -2.107 | 0.00125 |
| *NR1D2* | Nuclear receptor subfamily 1, group D, member 2 | -1.568 | 0.00205 |
| *NR3C2* | Nuclear receptor subfamily 3, group C, member 2 | -1.647 | 0.00258 |
| *NRG2* | Neuregulin 2 | -1.474 | 0.00298 |
| *NRROS* | Negative regulator of reactive oxygen species | 1.404 | 0.00488 |
| *NT5DC1* | 5'-nucleotidase domain containing 1 | -1.977 | 4.58E-13 |
| *NUAK1* | NUAK family, SNF1-like kinase, 1 | -1.635 | 0.00477 |
| *NUDT19* | Nudix (nucleoside diphosphate linked moiety X)-type motif 19 | -1.442 | 0.00142 |
| *NUDT8* | Nudix (nucleoside diphosphate linked moiety X)-type motif 8 | 1.363 | 0.00212 |
| *NUF2* | NUF2, NDC80 kinetochore complex component | -3.728 | 2.16E-06 |
| *NUGGC* | Nuclear GTPase, germinal center associated | -1.357 | 0.0001 |
| *NUP210* | Nucleoporin 210kDa | -1.257 | 0.00393 |
| *NXT2* | Nuclear transport factor 2-like export factor 2 | 1.456 | 0.00016 |
| *OAT* | Ornithine aminotransferase | -2.942 | 3.31E-11 |
| *OGDH* | Oxoglutarate (alpha-ketoglutarate) dehydrogenase (lipoamide) | -1.413 | 0.00046 |
| *ONECUT1* | One cut homeobox 1 | -1.433 | 0.00255 |
| *ORMDL3* | ORM1-like 3 (S. cerevisiae) | -1.328 | 0.0012 |
| *OSBPL11* | Oxysterol binding protein-like 11 | 1.342 | 0.00316 |
| *OTUD6B* | OTU domain containing 6B | -1.332 | 0.00382 |
| *P4HTM* | Prolyl 4-hydroxylase, transmembrane (endoplasmic reticulum) | 1.478 | 0.00472 |
| *PAM16* | Presequence translocase-associated motor 16 homolog (S. cerevisiae) | 1.436 | 0.00012 |
| *PANX1* | Pannexin 1 | -1.674 | 3.57E-05 |
| *PAPD7* | PAP associated domain containing 7 | 1.401 | 9.74E-05 |
| *PAPSS2* | 3'-phosphoadenosine 5'-phosphosulfate synthase 2 | -1.394 | 0.00142 |
| *PARVA* | Parvin, alpha | -1.429 | 2.34E-06 |
| *PATZ1* | POZ (BTB) and AT hook containing zinc finger 1 | 1.509 | 4.08E-05 |
| *PC* | Pyruvate carboxylase | 2.039 | 3.10E-14 |
| *PCCB* | Propionyl CoA carboxylase, beta polypeptide | -1.285 | 0.00276 |
| *PCDH1* | Protocadherin 1 | -1.363 | 0.00101 |
| *PCDH19* | Protocadherin 19 | -9.477 | 2.86E-16 |
| *PCED1A* | PC-esterase domain containing 1A | 1.427 | 0.00025 |
| *PCSK5* | Proprotein convertase subtilisin/kexin type 5 | -1.498 | 0.0022 |
| *PCSK6* | Proprotein convertase subtilisin/kexin type 6 | -1.386 | 0.00021 |
| *PCYOX1* | Prenylcysteine oxidase 1 | -1.659 | 1.7E-05 |
| *PDE3B* | Phosphodiesterase 3B, cGMP-inhibited | -1.526 | 4.54E-05 |
| *PDE4D* | Phosphodiesterase 4D, cAMP-specific | -1.661 | 0.00102 |
| *PDE4DIP* | Phosphodiesterase 4D interacting protein | -2.344 | 1.28E-13 |
| *PDE6C* | Phosphodiesterase 6C, cGMP-specific, cone, alpha prime | 1.466 | 0.00078 |
| *PDE8B* | Phosphodiesterase 8B | -1.516 | 0.00197 |
| *PDK2* | Pyruvate dehydrogenase kinase, isozyme 2 | -1.442 | 0.00012 |
| *PDLIM1* | PDZ and LIM domain 1 | 1.601 | 4.70E-06 |
| *PDLIM2* | PDZ and LIM domain 2 (mystique) | 1.471 | 0.00019 |
| *PDPR* | Ppyruvate dehydrogenase phosphatase regulatory subunit | -1.454 | 0.00087 |
| *PDXK* | Pyridoxal (pyridoxine, vitamin B6) kinase | -1.463 | 0.00012 |
| *PDZK1IP1* | PDZK1 interacting protein 1 | 2.309 | 0.00226 |
| *PEX5* | Peroxisomal biogenesis factor 5 | -1.383 | 4.46E-05 |
| *PFAS* | Phosphoribosylformylglycinamidine synthase | -1.332 | 0.00222 |
| *PFDN5* | Prefoldin subunit 5 | 1.326 | 0.00027 |
| *PGAP1* | Post-GPI attachment to proteins 1 | -2.122 | 0.00041 |
| *PGD* | Phosphogluconate dehydrogenase | 1.414 | 9E-06 |
| *PGM2* | Phosphoglucomutase 2 | -1.504 | 0.00025 |
| *PHAX* | Phosphorylated adaptor for RNA export | 1.279 | 0.00073 |
| *PHLPP1* | PH domain and leucine rich repeat protein phosphatase 1 | -1.382 | 0.00014 |
| *PI16* | Peptidase inhibitor 16 | -2.073 | 0.00073 |
| *PIGL* | Phosphatidylinositol glycan anchor biosynthesis, class L | 1.45 | 0.00014 |
| *PIK3C2G* | Phosphatidylinositol-4-phosphate 3-kinase, catalytic subunit type 2 gamma | -1.293 | 0.0014 |
| *PIK3R1* | Phosphoinositide-3-kinase, regulatory subunit 1 (alpha) | -1.47 | 2.42E-06 |
| *PIR* | Pirin (iron-binding nuclear protein) | 1.269 | 0.00144 |
| *PKIA* | Protein kinase (cAMP-dependent, catalytic) inhibitor alpha | -1.772 | 4.67E-06 |
| *PKN2* | Protein kinase N2 | -1.289 | 0.00517 |
| *PLCB1* | Phospholipase C, beta 1 (phosphoinositide-specific) | -1.434 | 0.00032 |
| *PLCD4* | Phospholipase C, delta 4 | -3.859 | 6.71E-22 |
| *PLCH1* | Phospholipase C, eta 1 | -1.915 | 0.00016 |
| *PLD1* | Phospholipase D1, phosphatidylcholine-specific | -1.797 | 8.03E-09 |
| *PLEC* | Plectin | -1.612 | 6.60E-06 |
| *PLEKHA4* | Pleckstrin homology domain containing, family A (phosphoinositide binding specific) member 4 | 1.623 | 0.00117 |
| *PLEKHA7* | Pleckstrin homology domain containing, family A member 7 | -1.324 | 0.0017 |
| *PLIN5* | Perilipin 5 | 1.588 | 0.00091 |
| *PLK2* | Polo-like kinase 2 | 1.359 | 0.00056 |
| *PLK3* | Polo-like kinase 3 | 2.155 | 6.14E-07 |
| *PLP2* | Proteolipid protein 2 (colonic epithelium-enriched) | 1.385 | 0.00538 |
| *PLRG1* | Pleiotropic regulator 1 | -1.292 | 0.00117 |
| *PLXNA4* | Plexin A4 | -2.091 | 1.78E-05 |
| *PMM2* | Phosphomannomutase 2 | 1.299 | 0.00104 |
| *PNPO* | Pyridoxamine 5'-phosphate oxidase | -1.479 | 0.00041 |
| *POLE* | Polymerase (DNA directed), epsilon, catalytic subunit | -1.486 | 0.00439 |
| *POLG* | Polymerase (DNA directed), gamma | -1.276 | 0.00012 |
| *POMC* | Proopiomelanocortin | 1.591 | 0.00503 |
| *PON1* | Paraoxonase 1 | 1.667 | 0.00382 |
| *POP1* | Processing of precursor 1, ribonuclease P/MRP subunit (S. cerevisiae) | 1.582 | 0.00336 |
| *PPA1* | Pyrophosphatase (inorganic) 1 | -1.618 | 8.38E-09 |
| *PPARA* | Peroxisome proliferator-activated receptor alpha | -1.364 | 0.00241 |
| *PPIP5K2* | Diphosphoinositol pentakisphosphate kinase 2 | -1.689 | 2.27E-07 |
| *PPL* | Periplakin | -1.603 | 0.00018 |
| *PPOX* | Protoporphyrinogen oxidase | 1.344 | 3.06E-05 |
| *PPP1R10* | Protein phosphatase 1, regulatory subunit 10 | -1.644 | 1.15E-05 |
| *PPP1R1B* | Protein phosphatase 1, regulatory (inhibitor) subunit 1B | -2.604 | 0.00033 |
| *PPP1R3B* | Protein phosphatase 1, regulatory subunit 3B | -1.645 | 0.00012 |
| *PPP2R3A* | Protein phosphatase 2, regulatory subunit B'', alpha | -1.314 | 0.00268 |
| *PPTC7* | PTC7 protein phosphatase homolog (S. cerevisiae) | -1.637 | 0.00276 |
| *PQLC1* | PQ loop repeat containing 1 | -1.352 | 0.00058 |
| *PRAP1* | Proline-rich acidic protein 1 | 8.633 | 6.58E-10 |
| *PRDM15* | PR domain containing 15 | -2.033 | 1.70E-06 |
| *PRDX1* | Peroxiredoxin 1 | 1.293 | 0.00119 |
| *PREB* | Prolactin regulatory element binding | 1.33 | 0.00017 |
| *PRF1* | Perforin 1 (pore forming protein) | 2.179 | 4.76E-05 |
| *PRKAR2A* | Protein kinase, cAMP-dependent, regulatory, type II, alpha | -1.29 | 0.0054 |
| *PRKCA* | Protein kinase C, alpha | -1.422 | 0.00244 |
| *PRKD3* | Protein kinase D3 | -1.302 | 0.00285 |
| *PRODH2* | Proline dehydrogenase (oxidase) 2 | -1.746 | 2.18E-06 |
| *PROSER2* | Proline and serine rich 2 | 1.431 | 1.81E-05 |
| *PROX2* | Prospero homeobox 2 | -2.884 | 0.00053 |
| *PRR16* | Proline rich 16 | 1.975 | 3.01E-05 |
| *PRSS23* | Protease, serine, 23 | -1.345 | 0.00192 |
| *PSD3* | Pleckstrin and Sec7 domain containing 3 | -1.961 | 2.68E-06 |
| *PSMB3* | Proteasome (prosome, macropain) subunit, beta type, 3 | 1.266 | 0.00182 |
| *PSMB8* | Proteasome (prosome, macropain) subunit, beta type, 8 | 1.339 | 0.00196 |
| *PSMG4* | Proteasome (prosome, macropain) assembly chaperone 4 | 1.711 | 5.46E-07 |
| *PSPH* | Phosphoserine phosphatase | 3.674 | 1.61E-09 |
| *PSTPIP1* | Proline-serine-threonine phosphatase interacting protein 1 | 1.641 | 0.00202 |
| *PTEN* | Phosphatase and tensin homolog | -1.499 | 6E-06 |
| *PTGFRN* | Prostaglandin F2 receptor inhibitor | -1.572 | 0.00012 |
| *PTGR1* | Prostaglandin reductase 1 | 1.773 | 4.09E-08 |
| *PTPN21* | Protein tyrosine phosphatase, non-receptor type 21 | -1.364 | 0.0009 |
| *PTPN7* | Protein tyrosine phosphatase, non-receptor type 7 | 1.719 | 0.00318 |
| *PTPN9* | Protein tyrosine phosphatase, non-receptor type 9 | -1.375 | 0.00109 |
| *PTPRCAP* | Protein tyrosine phosphatase, receptor type, C-associated protein | 1.642 | 0.00154 |
| *PTX3* | Pentraxin 3, long | 2.856 | 0.00578 |
| *PVRIG* | Poliovirus receptor related immunoglobulin domain containing | 1.883 | 0.00373 |
| *PYCR1* | Pyrroline-5-carboxylate reductase 1 | 9.465 | 2.31E-32 |
| *PYGL* | Phosphorylase, glycogen, liver | -1.45 | 9.1E-05 |
| *QKI* | QKI, KH domain containing, RNA binding | -1.467 | 7.72E-07 |
| *RAB29* | RAB29, member RAS oncogene family | 1.392 | 0.00088 |
| *RAB40B* | RAB40B, member RAS oncogene family | -1.769 | 3.92E-07 |
| *RABAC1* | Rab acceptor 1 (prenylated) | 1.284 | 0.00065 |
| *RAD21L1* | RAD21-like 1 (S. pombe) | -1.729 | 1.98E-07 |
| *RAD23A* | RAD23 homolog A (S. cerevisiae) | 1.265 | 0.00031 |
| *RAD54B* | RAD54 homolog B (S. cerevisiae) | -1.675 | 1.04E-05 |
| *RAMP3* | Receptor (G protein-coupled) activity modifying protein 3 | 1.348 | 0.00078 |
| *RANBP1* | RAN binding protein 1 | 1.323 | 0.00373 |
| *RAP1GAP* | RAP1 GTPase activating protein | 1.446 | 0.00504 |
| *RARG* | Retinoic acid receptor, gamma | 1.74 | 0.00013 |
| *RARRES2* | Retinoic acid receptor responder (tazarotene induced) 2 | 1.359 | 0.00097 |
| *RASAL3* | RAS protein activator like 3 | 1.764 | 0.00232 |
| *RASEF* | RAS and EF-hand domain containing | -5.119 | 1.91E-18 |
| *RASGRF2* | Ras protein-specific guanine nucleotide-releasing factor 2 | -2.653 | 0.0016 |
| *RASGRP2* | RAS guanyl releasing protein 2 (calcium and DAG-regulated) | 1.379 | 0.00512 |
| *RASSF6* | Ras association (RalGDS/AF-6) domain family member 6 | -2.012 | 4.14E-11 |
| *RBBP8* | Retinoblastoma binding protein 8 | -1.344 | 0.00055 |
| *RBM3* | RNA binding motif (RNP1, RRM) protein 3 | 1.496 | 0.00364 |
| *RBM47* | RNA binding motif protein 47 | -1.461 | 0.00155 |
| *RC3H2* | Ring finger and CCCH-type domains 2 | -1.462 | 0.00146 |
| *RCL1* | RNA terminal phosphate cyclase-like 1 | 1.315 | 0.00507 |
| *RENBP* | Renin binding protein | 1.532 | 4.46E-05 |
| *REPIN1* | Replication initiator 1 | 1.398 | 0.00216 |
| *RETSAT* | Retinol saturase (all-trans-retinol 13,14-reductase) | -2.183 | 2.18E-11 |
| *REV3L* | REV3-like, polymerase (DNA directed), zeta, catalytic subunit | -1.39 | 0.00149 |
| *RFX5* | Rregulatory factor X, 5 (influences HLA class II expression) | -1.727 | 7.55E-06 |
| *RFXANK* | Regulatory factor X-associated ankyrin-containing protein | 1.357 | 0.00204 |
| *RGS1* | Regulator of G-protein signaling 1 | 1.959 | 0.00163 |
| *RGS5* | Regulator of G-protein signaling 5 | -1.929 | 4.17E-05 |
| *RHEB* | Ras homolog enriched in brain | 1.293 | 0.00539 |
| *RHOC* | Ras homolog family member C | -1.263 | 0.00275 |
| *RHPN2* | Rhophilin, Rho GTPase binding protein 2 | -1.347 | 0.00016 |
| *RILPL2* | Rab interacting lysosomal protein-like 2 | -2.127 | 1.79E-08 |
| *RNASEH2C* | Ribonuclease H2, subunit C | 1.271 | 0.00508 |
| *ROBO1* | Roundabout, axon guidance receptor, homolog 1 (Drosophila) | -1.555 | 0.00082 |
| *ROBO2* | Roundabout guidance receptor 2 | -2.508 | 0.00077 |
| *RORC* | RAR-related orphan receptor C | 1.438 | 0.00014 |
| *Rpl10* | Ribosomal protein L10 | 1.389 | 0.00012 |
| *RPL10A* | Ribosomal protein L10a | 1.272 | 0.00121 |
| *RPL11* | Ribosomal protein L11 | 1.279 | 0.00076 |
| *RPL13* | Ribosomal protein L13 | 1.438 | 7.14E-05 |
| *RPL13A* | Ribosomal protein L13a | 1.358 | 0.00032 |
| *RPL14* | Ribosomal protein L14 | 1.489 | 1.72E-05 |
| *RPL15* | Ribosomal protein L15 | 1.338 | 7.81E-05 |
| *RPL17* | Ribosomal protein L17 | 1.369 | 3.11E-05 |
| *RPL18* | Ribosomal protein L18 | 1.341 | 0.0006 |
| *RPL18A* | Ribosomal protein L18a | 1.503 | 4.78E-06 |
| *RPL21* | Ribosomal protein L21 | 1.295 | 0.00077 |
| *RPL22* | Ribosomal protein L22 | 1.405 | 2.26E-05 |
| *RPL23* | Ribosomal protein L23 | 1.366 | 9.43E-05 |
| *RPL23A* | Ribosomal protein L23a | 1.287 | 0.00048 |
| *RPL24* | Ribosomal protein L24 | 1.286 | 0.00065 |
| *RPL26* | Ribosomal protein L26 | 1.299 | 8.74E-05 |
| *RPL27* | Ribosomal protein L27 | 1.309 | 0.00036 |
| *RPL27A* | Ribosomal protein L27a | 1.298 | 0.00081 |
| *RPL28* | Ribosomal protein L28 | 1.356 | 0.0008 |
| *RPL29* | Ribosomal protein L29 | 1.502 | 5.33E-07 |
| *RPL31* | Ribosomal protein L31 | 1.459 | 8.05E-05 |
| *RPL32* | Ribosomal protein L32 | 1.379 | 2.62E-05 |
| *RPL35* | Ribosomal protein L35 | 1.614 | 6.38E-09 |
| *RPL35A* | Ribosomal protein L35a | 1.351 | 0.00035 |
| *RPL36* | Ribosomal protein L36 | 1.697 | 3.76E-10 |
| *RPL36A* | Ribosomal protein L36a | 1.342 | 0.00168 |
| *RPL37* | Ribosomal protein L37 | 1.475 | 4.94E-07 |
| *RPL37A* | Ribosomal protein L37a | 1.627 | 7.00E-09 |
| *RPL38* | Ribosomal protein L38 | 1.624 | 1.47E-08 |
| *RPL5* | Ribosomal protein L5 | 1.314 | 0.00016 |
| *RPL6* | Ribosomal protein L6 | 1.304 | 7.73E-05 |
| *RPL7A* | Ribosomal protein L7a | 1.419 | 2.07E-05 |
| *RPL8* | Ribosomal protein L8 | 1.391 | 1.23E-05 |
| *RPLP1* | Ribosomal protein, large, P1 | 1.29 | 0.0111 |
| *RPLP2* | Ribosomal protein, large, P2 | 1.463 | 1.35E-05 |
| *RPS10* | Ribosomal protein S10 | 1.372 | 3.23E-05 |
| *RPS11* | Ribosomal protein S11 | 1.405 | 5.82E-05 |
| *RPS12* | Ribosomal protein S12 | 1.275 | 0.00148 |
| *RPS13* | Ribosomal protein S13 | 1.309 | 0.00019 |
| *RPS14* | Ribosomal protein S14 | 1.38 | 0.00011 |
| *RPS15* | Ribosomal protein S15 | 1.449 | 4.73E-06 |
| *RPS15A* | Ribosomal protein S15a | 1.275 | 0.00042 |
| *RPS16* | Ribosomal protein S16 | 1.372 | 3.13E-05 |
| *RPS18* | Ribosomal protein S18 | 1.475 | 1.92E-05 |
| *RPS19* | Ribosomal protein S19 | 1.389 | 4.2E-05 |
| *RPS20* | Ribosomal protein S20 | 1.389 | 5.02E-05 |
| *RPS21* | Ribosomal protein S21 | 1.345 | 0.00082 |
| *RPS23* | Ribosomal protein S23 | 1.544 | 5.48E-05 |
| *RPS24* | Ribosomal protein S24 | 1.388 | 2.97E-05 |
| *RPS25* | Ribosomal protein S25 | 1.38 | 5.91E-05 |
| *RPS27* | Ribosomal protein S27 | 1.573 | 1.98E-08 |
| *RPS27A* | Ribosomal protein S27a | 1.281 | 0.00162 |
| *RPS28* | Ribosomal protein S28 | 1.49 | 9.30E-06 |
| *RPS29* | Ribosomal protein S29 | 1.488 | 3.48E-06 |
| *RPS3* | Ribosomal protein S3 | 1.317 | 0.00109 |
| *RPS3A* | Ribosomal protein S3A | 1.267 | 0.00227 |
| *RPS4X* | Ribosomal protein S4, X-linked | 1.281 | 0.00023 |
| *RPS4Y2* | Ribosomal protein S4, Y-linked 2 | 1.281 | 0.00023 |
| *RPS5* | Ribosomal protein S5 | 1.432 | 4.82E-05 |
| *RPS6* | Ribosomal protein S6 | 1.362 | 1.02E-05 |
| *RPS7* | Ribosomal protein S7 | 1.409 | 0.00015 |
| *RPS8* | Ribosomal protein S8 | 1.48 | 3.62E-05 |
| *RPS9* | Ribosomal protein S9 | 1.395 | 0.00026 |
| *RRAGD* | Ras-related GTP binding D | 1.802 | 0.00042 |
| *RRP9* | Ribosomal RNA processing 9, small subunit (SSU) processome component, homolog (yeast) | 1.373 | 0.00277 |
| *RSPO3* | R-spondin 3 | 1.659 | 3.07E-05 |
| *RTKN* | Rhotekin | 1.527 | 0.00171 |
| *RTN1* | Reticulon 1 | 1.486 | 0.00047 |
| *RTN2* | Reticulon 2 | 1.659 | 4.59E-08 |
| *RUSC2* | RUN and SH3 domain containing 2 | 1.635 | 0.00021 |
| *RXRG* | Retinoid X receptor, gamma | 2.609 | 9.58E-07 |
| *S100A1* | S100 calcium binding protein A1 | 2.365 | 4.72E-06 |
| *S100A13* | S100 calcium binding protein A13 | 1.848 | 6.18E-08 |
| *S100PBP* | S100P binding protein | -1.379 | 0.00067 |
| *SAMD9* | Sterile alpha motif domain containing 9 | 1.514 | 0.00512 |
| *SAP130* | Sin3A-associated protein, 130kDa | -1.335 | 5.45E-05 |
| *SAR1B* | SAR1 homolog B (S. cerevisiae) | -1.363 | 0.00286 |
| *SARDH* | Sarcosine dehydrogenase | -1.383 | 0.00156 |
| *SCAND1* | SCAN domain containing 1 | 1.543 | 3.23E-05 |
| *SCFD2* | Sec1 family domain containing 2 | -1.446 | 1.63E-06 |
| *SCGB1D2* | Secretoglobin, family 1D, member 2 | 2.143 | 3.6E-05 |
| *SCIN* | Scinderin | -2.01 | 0.00201 |
| *SCRN2* | Secernin 2 | 1.377 | 0.00166 |
| *SDHA* | Succinate dehydrogenase complex, subunit A, flavoprotein (Fp) | -1.533 | 4.75E-07 |
| *SDHC* | Succinate dehydrogenase complex, subunit C, integral membrane protein, 15kDa | -1.36 | 0.00029 |
| *SEC14L3* | SEC14-like 3 (S. cerevisiae) | -1.791 | 3.29E-05 |
| *SEC16B* | SEC16 homolog B (S. cerevisiae) | -1.716 | 3.79E-08 |
| *SEC61G* | Sec61 gamma subunit | 1.394 | 0.00194 |
| *Sectm1b* | Secreted and transmembrane 1B | -2.272 | 0.00453 |
| *SELK* | Selenoprotein K | 1.507 | 1.00E-07 |
| *SELM* | Selenoprotein M | 1.641 | 0.00466 |
| *SELO* | Selenoprotein O | 1.432 | 0.00062 |
| *SEMA4B* | Sema domain, immunoglobulin domain (Ig), transmembrane domain (TM) and short Cytoplasmic domain, (semaphorin) 4B | -1.458 | 1.88E-05 |
| *SEMA5A* | sema domain, seven thrombospondin repeats (type 1 and type 1-like), transmembrane domain (TM) and short cytoplasmic domain, (semaphorin) 5A | -1.627 | 0.0013 |
| *SEPT.1* | Septin 1 | 1.675 | 0.00135 |
| *SEPT.11* | Septin 11 | -1.369 | 0.00147 |
| *SERPINA1* | Serpin peptidase inhibitor, clade A (alpha-1 antiproteinase, antitrypsin), member 1 | 1.505 | 0.0013 |
| *SESN3* | Sestrin 3 | -1.969 | 3.71E-09 |
| *SETX* | Senataxin | -1.294 | 0.00078 |
| *SFMBT2* | Scm-like with four mbt domains 2 | -1.835 | 1.59E-07 |
| *SFRP1* | Secreted frizzled-related protein 1 | -2.889 | 4.96E-05 |
| *SFT2D2* | SFT2 domain containing 2 | -1.575 | 0.00039 |
| *SGK2* | Serum/glucocorticoid regulated kinase 2 | -1.736 | 8.09E-11 |
| *SGMS1* | Sphingomyelin synthase 1 | -1.375 | 0.0029 |
| *SGMS2* | Sphingomyelin synthase 2 | -1.919 | 0.00021 |
| *SH2D1A* | SH2 domain containing 1A | 2.033 | 0.00125 |
| *SH3D19* | SH3 domain containing 19 | -1.54 | 1.54E-06 |
| *SH3PXD2B* | SH3 and PX domains 2B | -1.625 | 9.85E-05 |
| *SHPK* | Sedoheptulokinase | -1.467 | 3.63E-05 |
| *SHTN1* | Shootin 1 | 1.256 | 0.00374 |
| *SIAH1* | Siah E3 ubiquitin protein ligase 1 | 1.377 | 0.00014 |
| *SIAH1* | Siah E3 ubiquitin protein ligase 1 | 1.377 | 0.00014 |
| *SIRT2* | Sirtuin 2 | 1.349 | 0.0005 |
| *SLA2* | Src-like-adaptor 2 | -1.41 | 0.00254 |
| *SLC16A12* | Solute carrier family 16, member 12 | -1.712 | 1.09E-06 |
| *SLC16A5* | Solute carrier family 16 (monocarboxylate transporter), member 5 | 1.959 | 0.00018 |
| *SLC16A6* | Solute carrier family 16, member 6 | -2.091 | 3.23E-10 |
| *SLC17A4* | Solute carrier family 17, member 4 | -1.444 | 0.00266 |
| *SLC17A9* | Solute carrier family 17 (vesicular nucleotide transporter), member 9 | 1.676 | 0.00515 |
| *SLC19A1* | Solute carrier family 19 (folate transporter), member 1 | 1.669 | 0.00099 |
| *SLC19A2* | Solute carrier family 19 (thiamine transporter), member 2 | -1.409 | 0.00272 |
| *SLC1A4* | Solute carrier family 1 (glutamate/neutral amino acid transporter), member 4 | 1.612 | 4.80E-06 |
| *SLC20A2* | Solute carrier family 20 (phosphate transporter), member 2 | -1.323 | 0.00205 |
| *SLC22A10* | Solute carrier family 22, member 10 | -1.883 | 3.71E-07 |
| *SLC23A1* | Solute carrier family 23 (ascorbic acid transporter), member 1 | -1.404 | 0.00024 |
| *SLC25A15* | Solute carrier family 25 (mitochondrial carrier; ornithine transporter) member 15 | -1.478 | 0.00133 |
| *SLC25A20* | Solute carrier family 25 (carnitine/acylcarnitine translocase), member 20 | -1.357 | 0.00027 |
| *SLC25A33* | Solute carrier family 25 (pyrimidine nucleotide carrier), member 33 | 2.064 | 1.14E-08 |
| *SLC25A6* | Solute carrier family 25 (mitochondrial carrier; adenine nucleotide translocator), member 6 | 1.299 | 0.0053 |
| *SLC26A11* | Solute carrier family 26 (anion exchanger), member 11 | -1.694 | 4.43E-08 |
| *SLC26A7* | Solute carrier family 26 (anion exchanger), member 7 | -1.897 | 0.00063 |
| *SLC27A4* | Solute carrier family 27 (fatty acid transporter), member 4 | -2.112 | 1.21E-12 |
| *SLC2A5* | Solute carrier family 2 (facilitated glucose/fructose transporter), member 5 | -9.346 | 1.74E-15 |
| *SLC30A10* | Solute carrier family 30, member 10 | -1.677 | 0.00013 |
| *SLC30A6* | Solute carrier family 30 (zinc transporter), member 6 | -1.317 | 0.00204 |
| *SLC35D2* | Solute carrier family 35 (UDP-GlcNAc/UDP-glucose transporter), member D2 | -1.253 | 0.00523 |
| *SLC36A1* | Solute carrier family 36 (proton/amino acid symporter), member 1 | -1.581 | 0.00118 |
| *SLC37A4* | Solute carrier family 37 (glucose-6-phosphate transporter), member 4 | -1.328 | 8.92E-05 |
| *SLC38A2* | Solute carrier family 38, member 2 | 1.439 | 0.00556 |
| *SLC38A4* | Solute carrier family 38, member 4 | -1.433 | 0.00312 |
| *SLC39A9* | Solute carrier family 39, member 9 | -1.29 | 0.00494 |
| *SLC41A2* | Solute carrier family 41 (magnesium transporter), member 2 | -1.489 | 0.00071 |
| *SLC44A1* | Solute carrier family 44 (choline transporter), member 1 | -1.748 | 3.69E-07 |
| *SLC48A1* | Solute carrier family 48 (heme transporter), member 1 | 1.399 | 1.99E-06 |
| *SLC4A4* | Solute carrier family 4 (sodium bicarbonate cotransporter), member 4 | -1.719 | 9.37E-07 |
| *SLC51B* | Solute carrier family 51, beta subunit | -1.561 | 2.86E-05 |
| *SLC5A1* | Solute carrier family 5 (sodium/glucose cotransporter), member 1 | -1.852 | 0.00003 |
| *SLC6A14* | Solute carrier family 6 (amino acid transporter), member 14 | 1.541 | 0.016 |
| *SLC7A2* | Solute carrier family 7 (cationic amino acid transporter, y+ system), member 2 | -2.853 | 3.98E-09 |
| *SLC7A9* | Solute carrier family 7 (amino acid transporter light chain, bo,+ system), member 9 | -1.427 | 0.0043 |
| *SLCO1A2* | Solute carrier organic anion transporter family, member 1A2 | -1.539 | 0.00029 |
| *SLCO4C1* | Solute carrier organic anion transporter family, member 4C1 | -1.737 | 6.24E-06 |
| *SLFN11* | Schlafen family member 11 | -1.674 | 0.00021 |
| *SLIRP* | SRA stem-loop interacting RNA binding protein | 1.32 | 0.00014 |
| *SMC1A* | Structural maintenance of chromosomes 1A | -1.467 | 0.00048 |
| *SMCR8* | Smith-Magenis syndrome chromosome region, candidate 8 | -1.37 | 0.00398 |
| *SMPD3* | Sphingomyelin phosphodiesterase 3, neutral membrane (neutral sphingomyelinase II) | -1.366 | 0.00164 |
| *SMPDL3B* | Sphingomyelin phosphodiesterase, acid-like 3B | -1.871 | 6.92E-05 |
| *SNCAIP* | Synuclein, alpha interacting protein | -1.788 | 1.36E-05 |
| *SNRPG* | Small nuclear ribonucleoprotein polypeptide G | 1.412 | 0.00208 |
| *SNTB1* | Syntrophin, beta 1 (dystrophin-associated protein A1, 59kDa, basic component 1) | 1.303 | 0.00551 |
| *SNX10* | Sorting nexin 10 | 1.352 | 0.00049 |
| *SOCS2* | Suppressor of cytokine signaling 2 | 1.659 | 0.00503 |
| *SOD2* | Superoxide dismutase 2, mitochondrial | -1.411 | 0.00117 |
| *SOD3* | Superoxide dismutase 3, extracellular | -6.333 | 1.16E-12 |
| *SORBS1* | Sorbin and SH3 domain containing 1 | -1.421 | 0.00166 |
| *SOWAHB* | Sosondowah ankyrin repeat domain family member B | -1.573 | 0.00051 |
| *SPATA13* | Spermatogenesis associated 13 | -1.315 | 0.00257 |
| *SPEF2* | Sperm flagellar 2 | 2.399 | 1.47E-05 |
| *SPNS2* | Spinster homolog 2 (Drosophila) | -1.709 | 0.00025 |
| *SPP1* | Secreted phosphoprotein 1 | -4.257 | 1.72E-05 |
| *SPR* | Sepiapterin reductase (7,8-dihydrobiopterin:NADP+ oxidoreductase) | 1.258 | 0.0005 |
| *SPTLC3* | Serine palmitoyltransferase, long chain base subunit 3 | -1.719 | 8.5E-05 |
| *SPX* | Spexin hormone | 2.297 | 2.04E-06 |
| *SRA1* | Steroid receptor RNA activator 1 | 1.282 | 0.00358 |
| *SRC* | V-src avian sarcoma (Schmidt-Ruppin A-2) viral oncogene homolog | -1.654 | 0.0016 |
| *SRXN1* | Sulfiredoxin 1 | 2.37 | 2.33E-06 |
| *SSR4* | Signal sequence receptor, delta | 1.25 | 0.0055 |
| *ST3GAL4* | ST3 beta-galactoside alpha-2,3-sialyltransferase 4 | 1.363 | 0.00039 |
| *STARD9* | StAR-related lipid transfer (START) domain containing 9 | 1.545 | 0.00448 |
| *STEAP4* | STEAP family member 4 | 1.847 | 9.22E-05 |
| *STON2* | Stonin 2 | -1.558 | 0.00161 |
| *STRIP2* | Striatin interacting protein 2 | -3.115 | 1.34E-07 |
| *STS* | Steroid sulfatase (microsomal), isozyme S | -2.557 | 4.77E-05 |
| *Sult1a1* | Sulfotransferase family 1A, phenol-preferring, member 1 | -1.499 | 0.0001 |
| *SULT1B1* | Sulfotransferase family, cytosolic, 1B, member 1 | -1.488 | 0.00033 |
| *SULT1E1* | Sulfotransferase family 1E, estrogen-preferring, member 1 | 1.997 | 0.0014 |
| *SULT2A1* | Sulfotransferase family, cytosolic, 2A, dehydroepiandrosterone (DHEA)-preferring, member 1 | 1.363 | 0.00074 |
| *SWAP70* | SWAP switching B-cell complex 70kDa subunit | -1.33 | 0.0005 |
| *SWSAP1* | SWIM-type zinc finger 7 associated protein 1 | 1.444 | 0.00229 |
| *TANC1* | Tetratricopeptide repeat, ankyrin repeat and coiled-coil containing 1 | -1.427 | 0.00016 |
| *TANGO2* | Transport and golgi organization 2 homolog (Drosophila) | -1.366 | 0.00441 |
| *TARS* | Threonyl-tRNA synthetase | 1.262 | 0.00403 |
| *TAX1BP3* | Tax1 (human T-cell leukemia virus type I) binding protein 3 | -1.303 | 0.00013 |
| *TBATA* | Thymus, brain and testes associated | -2.308 | 0.00069 |
| *TBCEL* | Tubulin folding cofactor E-like | -1.415 | 0.00071 |
| *TBX21* | T-box 21 | 1.829 | 0.00428 |
| *TCEA2* | Transcription elongation factor A (SII), 2 | 2.079 | 7.22E-07 |
| *TCEB2* | Transcription elongation factor B (SIII), polypeptide 2 (18kDa, elongin B) | 1.336 | 0.00152 |
| *TCF21* | Transcription factor 21 | 1.41 | 0.00332 |
| *TCF7* | Transcription factor 7 (T-cell specific, HMG-box) | 1.889 | 3.99E-07 |
| *TCIRG1* | T-cell, immune regulator 1, ATPase, H+ transporting, lysosomal V0 subunit A3 | -1.409 | 0.00067 |
| *TCP11L2* | T-complex 11, testis-specific-like 2 | -1.271 | 0.00371 |
| *Tdh* | L-threonine dehydrogenase | -1.709 | 4.99E-05 |
| *TDO2* | Tryptophan 2,3-dioxygenase | -1.529 | 0.0002 |
| *TEAD1* | TEA domain family member 1 (SV40 transcriptional enhancer factor) | -1.577 | 0.00334 |
| *TEF* | Thyrotrophic embryonic factor | -1.412 | 0.00398 |
| *TENM3* | Teneurin transmembrane protein 3 | -2.068 | 4.94E-06 |
| *TESK1* | Testis-specific kinase 1 | 1.325 | 0.00238 |
| *TESK2* | Testis-specific kinase 2 | -1.371 | 0.00346 |
| *TEX12* | Testis expressed 12 | -2.875 | 2.06E-07 |
| *TEX2* | Testis expressed 2 | -1.374 | 0.00015 |
| *TF* | Transferrin | -1.737 | 0.0002 |
| *TFRC* | Transferrin receptor | -1.516 | 0.00172 |
| *TGOLN2* | Trans-golgi network protein 2 | -1.334 | 0.00155 |
| *THRSP* | Thyroid hormone responsive | -8.143 | 1.04E-26 |
| *TK2* | Thymidine kinase 2, mitochondrial | -1.3 | 0.00248 |
| *TLE4* | Transducin-like enhancer of split 4 (E(sp1) homolog, Drosophila) | -1.476 | 0.00022 |
| *TMEM120B* | Transmembrane protein 120B | -1.304 | 0.00309 |
| *TMEM160* | Transmembrane protein 160 | 1.591 | 0.00056 |
| *TMEM171* | Ttransmembrane protein 171 | -1.323 | 0.00502 |
| *TMEM181* | Transmembrane protein 181 | -1.295 | 0.0022 |
| *TMEM184C* | Transmembrane protein 184C | -1.508 | 1.54E-08 |
| *TMEM187* | Transmembrane protein 187 | -1.576 | 3.41E-06 |
| *TMEM19* | Transmembrane protein 19 | -1.317 | 0.00399 |
| *TMEM206* | Transmembrane protein 206 | 1.609 | 0.00037 |
| *TMEM25* | Transmembrane protein 25 | -1.483 | 0.00165 |
| *TMEM256* | Transmembrane protein 256 | 1.377 | 0.00031 |
| *TMEM42* | Transmembrane protein 42 | 1.444 | 0.00158 |
| *TMEM50B* | Transmembrane protein 50B | 1.398 | 5.97E-05 |
| *TMEM57* | Transmembrane protein 57 | -1.339 | 2.73E-05 |
| *TMEM63A* | Transmembrane protein 63A | -1.257 | 0.00169 |
| *TMEM86A* | Transmembrane protein 86A | 1.497 | 0.00017 |
| *TMEM8B* | Transmembrane protein 8B | 1.528 | 0.0008 |
| *TMF1* | TATA element modulatory factor 1 | -1.297 | 0.00136 |
| *TMPRSS7* | Transmembrane protease, serine 7 | -2.312 | 0.00033 |
| *TNFSF10* | Tumor necrosis factor (ligand) superfamily, member 10 | -2.496 | 3.83E-21 |
| *TNFSF13* | Tumor necrosis factor (ligand) superfamily, member 13 | 1.974 | 4.6E-05 |
| *TNFSF18* | Tumor necrosis factor (ligand) superfamily, member 18 | 2.452 | 2.24E-08 |
| *TNFSF9* | Tumor necrosis factor (ligand) superfamily, member 9 | 2.023 | 4.92E-05 |
| *TNKS* | Tankyrase, TRF1-interacting ankyrin-related ADP-ribose polymerase | -1.501 | 0.00124 |
| *TNKS2* | Tankyrase, TRF1-interacting ankyrin-related ADP-ribose polymerase 2 | -1.267 | 0.00482 |
| *TNPO1* | Transportin 1 | -1.291 | 0.00131 |
| *TOB1* | Transducer of ERBB2, 1 | -1.438 | 0.0002 |
| *TOMM6* | Translocase of outer mitochondrial membrane 6 homolog (yeast) | 1.328 | 0.00041 |
| *TOPBP1* | Topoisomerase (DNA) II binding protein 1 | -1.283 | 0.0014 |
| *TP53BP2* | Tumor protein p53 binding protein, 2 | -1.546 | 1.72E-06 |
| *TPT1* | Tumor protein, translationally-controlled 1 | 1.271 | 0.00544 |
| *TRAF3* | TNF receptor-associated factor 3 | -1.544 | 0.00189 |
| *TRAF3IP1* | TNF receptor-associated factor 3 interacting protein 1 | -1.339 | 0.00338 |
| *TRAPPC10* | Trafficking protein particle complex 10 | -1.311 | 0.00387 |
| *TRAPPC5* | Trafficking protein particle complex 5 | 1.425 | 0.00147 |
| *TRHDE* | Thyrotropin-releasing hormone degrading enzyme | -2.126 | 0.00515 |
| *TRIM24* | Tripartite motif containing 24 | -1.338 | 0.0004 |
| *TRIM32* | Tripartite motif containing 32 | 1.388 | 0.00096 |
| *TRIM47* | Tripartite motif containing 47 | 1.322 | 0.00408 |
| *TRIM7* | Tripartite motif containing 7 | 2.915 | 1.16E-06 |
| *TRMT112* | tRNA methyltransferase 11-2 homolog (S. cerevisiae) | 1.401 | 1.56E-05 |
| *TRMT13* | tRNA methyltransferase 13 homolog (S. cerevisiae) | 1.424 | 0.00195 |
| *TRMT61A* | tRNA methyltransferase 61 homolog A (S. cerevisiae) | 1.484 | 0.00111 |
| *TRPM8* | Transient receptor potential cation channel, subfamily M, member 8 | -1.919 | 1.68E-07 |
| *TSPAN13* | Tetraspanin 13 | -1.826 | 3.90E-11 |
| *TSPAN33* | Tetraspanin 33 | -2.292 | 1.48E-08 |
| *TTC17* | Tetratricopeptide repeat domain 17 | -1.296 | 0.00213 |
| *TTC23* | Tetratricopeptide repeat domain 23 | -1.628 | 0.00033 |
| *TTC32* | Tetratricopeptide repeat domain 32 | 1.375 | 0.00166 |
| *TTYH2* | Tweety family member 2 | -1.373 | 0.00137 |
| *TXN* | Thioredoxin | 1.627 | 2.54E-06 |
| *TXNDC16* | Thioredoxin domain containing 16 | -1.415 | 0.00531 |
| *TXNIP* | Thioredoxin interacting protein | 1.418 | 0.00223 |
| *TXNL4B* | Thioredoxin-like 4B | -1.417 | 3.62E-05 |
| *TXNRD1* | Thioredoxin reductase 1 | 1.276 | 0.0008 |
| *UAP1L1* | UDP-N-acteylglucosamine pyrophosphorylase 1-like 1 | 1.494 | 0.00042 |
| *UBA52* | Ubiquitin A-52 residue ribosomal protein fusion product 1 | 1.307 | 0.0041 |
| *UBD* | Ubiquitin D | 1.736 | 0.00013 |
| *UBL5* | Ubiquitin-like 5 | 1.336 | 0.0004 |
| *UBR1* | Ubiquitin protein ligase E3 component n-recognin 1 | -1.425 | 0.00107 |
| *UCHL1* | Ubiquitin carboxyl-terminal esterase L1 (ubiquitin thiolesterase) | 1.63 | 0.00231 |
| *UGCG* | UDP-glucose ceramide glucosyltransferase | -1.698 | 0.00013 |
| *UGDH* | UDP-glucose 6-dehydrogenase | 1.475 | 0.00366 |
| *UGT3A2* | UDP glycosyltransferase 3 family, polypeptide A2 | -1.542 | 0.00011 |
| *UNC13D* | Unc-13 homolog D (C. elegans) | 1.462 | 0.00092 |
| *UNC5A* | Unc-5 netrin receptor A | -1.949 | 3.80E-10 |
| *UNC5B* | Unc-5 homolog B (C. elegans) | -1.809 | 0.00024 |
| *UNC5C* | Unc-5 homolog C (C. elegans) | -2.259 | 0.00013 |
| *UNKL* | Unkempt family zinc finger-like | 1.532 | 0.00392 |
| *Uox* | Urate oxidase | 2.109 | 5.17E-06 |
| *UPP2* | Uridine phosphorylase 2 | -1.383 | 0.00519 |
| *UQCC1* | Ubiquinol-cytochrome c reductase complex assembly factor 1 | -1.306 | 4.03E-05 |
| *URAD* | Ureidoimidazoline (2-oxo-4-hydroxy-4-carboxy-5-) decarboxylase | -1.33 | 0.00484 |
| *USP2* | Ubiquitin specific peptidase 2 | -2.333 | 8.99E-05 |
| *USP46* | Ubiquitin specific peptidase 46 | -1.255 | 0.0032 |
| *UXT* | Ubiquitously-expressed, prefoldin-like chaperone | 1.356 | 8.37E-05 |
| *VAMP5* | Vesicle-associated membrane protein 5 | 1.388 | 0.00369 |
| *VCPIP1* | Valosin containing protein (p97)/p47 complex interacting protein 1 | -1.268 | 0.00139 |
| *Vegfb* | Vascular endothelial growth factor B | 1.793 | 2.91E-06 |
| *VPS41* | Vacuolar protein sorting 41 homolog (S. cerevisiae) | -1.262 | 0.00043 |
| *VRK2* | Vaccinia related kinase 2 | -1.358 | 0.00265 |
| *VWA8* | Von Willebrand factor A domain containing 8 | -1.634 | 8.19E-06 |
| *WDR89* | WD repeat domain 89 | 1.374 | 0.00549 |
| *WFDC2* | WAP four-disulfide core domain 2 | -6.461 | 0.00021 |
| *Wfdc21* | WAP four-disulfide core domain 21 | -2.147 | 0.00018 |
| *XAF1* | XIAP associated factor 1 | 1.639 | 7.13E-05 |
| *XDH* | Xanthine dehydrogenase | 1.447 | 9.15E-05 |
| *XK* | X-linked Kx blood group (McLeod syndrome) | -1.836 | 0.0002 |
| *XPNPEP2* | X-prolyl aminopeptidase (aminopeptidase P) 2, membrane-bound | -4.358 | 8.28E-22 |
| *YARS* | Tyrosyl-tRNA synthetase | 1.345 | 0.00027 |
| *YTHDF2* | YTH domain family, member 2 | -1.274 | 0.00429 |
| *ZAK* | Sterile alpha motif and leucine zipper containing kinase AZK | -1.492 | 0.00062 |
| *ZAP70* | Zeta-chain (TCR) associated protein kinase 70kDa | 1.698 | 2.85E-05 |
| *ZBTB10* | Zinc finger and BTB domain containing 10 | -1.689 | 7.11E-06 |
| *ZC3H12A* | Zinc finger CCCH-type containing 12A | 1.532 | 0.00025 |
| *ZC3H4* | Zinc finger CCCH-type containing 4 | -1.269 | 0.00561 |
| *ZC3HAV1* | Zinc finger CCCH-type, antiviral 1 | -1.298 | 0.00191 |
| *ZCCHC6* | Zinc finger, CCHC domain containing 6 | -1.464 | 7.89E-06 |
| *ZDHHC20* | Zinc finger, DHHC-type containing 20 | -1.473 | 0.00134 |
| *ZDHHC9* | Zinc finger, DHHC-type containing 9 | -1.357 | 0.00058 |
| *ZFAND2B* | Zinc finger, AN1-type domain 2B | 1.293 | 0.00122 |
| *ZFAND4* | Zinc finger, AN1-type domain 4 | -1.467 | 0.00062 |
| *ZKSCAN1* | Zinc finger with KRAB and SCAN domains 1 | -1.394 | 0.0003 |
| *ZMAT5* | Zinc finger, matrin-type 5 | 1.455 | 0.00214 |
| *ZNF106* | Zinc finger protein 106 | -1.322 | 0.00255 |
| *ZNF32* | Zinc finger protein 32 | 1.359 | 0.00207 |
| *ZNF358* | Zinc finger protein 358 | 1.464 | 0.00032 |
| *ZNF445* | Zinc finger protein 445 | -1.376 | 0.00429 |
| *ZNF516* | Zinc finger protein 516 | -1.824 | 0.00066 |
| *ZNF593* | Zinc finger protein 593 | 1.397 | 0.00468 |
| *ZNF667* | Zinc finger protein 667 | 1.338 | 0.00139 |
| *ZNHIT1* | Zinc finger, HIT-type containing 1 | 1.393 | 2.15E-05 |

^1^ Fold changes are up or down in restricted fed animals compared to *ad libitum* control animals
