# Supplementary material for: Effect of dietary restriction and subsequent re-alimentation on the transcriptional profile of hepatic tissue in cattle
Source: BMC Genomics. 2016 Mar 17;17:244. doi: 10.1186/s12864-016-2578-5 (PMC4794862; doi:10.1186/s12864-016-2578-5)
Supplement: Additional file 2: Table S2. — Differentially expressed genes following a period of compensatory growth in re-alimentation. (DOCX 17 kb) [file 12864_2016_2578_MOESM2_ESM.docx]

**Additional file 2: Table S2.** Differentially expressed genes following a period of compensatory growth in re-alimentation

| Symbol | Entrez Gene Name | Fold Change^1^ | p-value |
| --- | --- | --- | --- |
| *ARHGAP30* | Rho GTPase activating protein 30 | 1.578 | 0.0001 |
| *BCORL1* | BCL6 corepressor-like 1 | 1.539 | 0.00019 |
| *CBX4* | Chromobox homolog 4 | 1.817 | 0.00011 |
| *CCDC134* | Coiled-coil domain containing 134 | -2.772 | 1.7E-05 |
| *COL1A1* | Collagen, type I, alpha 1 | 4.797 | 1.6E-05 |
| *COL1A2* | Collagen, type I, alpha 2 | 3.421 | 0.00016 |
| *DDIT3* | DNA-damage-inducible transcript 3 | -1.976 | 5.1E-05 |
| *DDX58* | DEAD (Asp-Glu-Ala-Asp) box polypeptide 58 | 1.692 | 5.6E-05 |
| *DERL2* | Derlin 2 | -1.389 | 0.00014 |
| *DNAJB11* | DnaJ (Hsp40) homolog, subfamily B, member 11 | -2.211 | 0.00012 |
| *DNAJB9* | DnaJ (Hsp40) homolog, subfamily B, member 9 | -1.783 | 4.4E-05 |
| *EZR* | Ezrin | 1.459 | 0.0001 |
| *FADS1* | Fatty acid desaturase 1 | 2.702 | 1.3E-07 |
| *FBLN1* | Fibulin 1 | 3.145 | 3.5E-05 |
| *FKBP9* | FK506 binding protein 9, 63 kDa | 1.481 | 0.00011 |
| *GPBAR1* | G protein-coupled bile acid receptor 1 | 1.859 | 0.0001 |
| *HBD* | Hemoglobin, delta | -3.996 | 8.01E-07 |
| *HERPUD1* | Homocysteine-inducible, endoplasmic reticulum stress-inducible, ubiquitin-like domain member 1 | -2.041 | 0.00012 |
| *HNF1B* | HNF1 homeobox B | 1.722 | 7.7E-05 |
| *HSD3B2* | Hydroxy-delta-5-steroid dehydrogenase, 3 beta- and steroid delta-isomerase 2 | -1.913 | 2E-05 |
| *HSPA5* | Heat shock 70kDa protein 5 (glucose-regulated protein, 78kDa) | -2.326 | 0.0002 |
| *KRT7* | Keratin 7 | 2.2 | 0.0002 |
| *MANF* | Mesencephalic astrocyte-derived neurotrophic factor | -2.363 | 0.00013 |
| *MAST3* | Microtubule associated serine/threonine kinase 3 | 1.583 | 8.9E-06 |
| *MGP* | Matrix Gla protein | 2.325 | 8.5E-05 |
| *MIS12* | MIS12 kinetochore complex component | -1.645 | 9.8E-06 |
| *MX2* | Myxovirus (influenza virus) resistance 2 (mouse) | 2.556 | 0.00014 |
| *NUS1* | NUS1 dehydrodolichyl diphosphate synthase subunit | -1.444 | 7.00E-05 |
| *PARVG* | Parvin, gamma | 1.655 | 0.00016 |
| *PI16* | Peptidase inhibitor 16 | 3.339 | 5.24E-05 |
| *PPP1R1B* | Protein phosphatase 1, regulatory (inhibitor) subunit 1B | -2.214 | 2.7E-06 |
| *RPS27* | Rribosomal protein S27 | -1.296 | 0.00063 |
| *SELK* | Selenoprotein K | -1.683 | 1.1E-05 |
| *SEMA4B* | sema domain, immunoglobulin domain (Ig), transmembrane domain (TM) and short cytoplasmic domain, (semaphorin) 4B | 1.665 | 0.00013 |
| *SNRNP27* | Small nuclear ribonucleoprotein 27kDa (U4/U6.U5) | -1.466 | 0.00017 |
| *SPARC* | Secreted protein, acidic, cysteine-rich (osteonectin) | 2.115 | 0.00026 |
| *SREBF1* | Sterol regulatory element binding transcription factor 1 | 1.559 | 5.6E-05 |
| *TCEA3* | Transcription elongation factor A (SII), 3 | -1.945 | 3.6E-05 |
| *THAP5* | THAP domain containing 5 | -1.765 | 1.9E-05 |
| *TMEM220* | Transmembrane protein 220 | -1.848 | 2.45E-05 |
| *TMEM45A* | Transmembrane protein 45A | -7.666 | 3.5E-06 |

^1^ Fold changes are up or down in restricted fed animals compared to *ad libitum* control animals
