# Supplementary material for: Effect of dietary restriction and subsequent re-alimentation on the transcriptional profile of hepatic tissue in cattle
Source: BMC Genomics. 2016 Mar 17;17:244. doi: 10.1186/s12864-016-2578-5 (PMC4794862; doi:10.1186/s12864-016-2578-5)
Supplement: Additional file 3: Table S3. — Networks generated from gene expression data of restricted versus ad libitum fed bulls by IPA. (DOCX 21 kb) [file 12864_2016_2578_MOESM3_ESM.docx]

**Additional file 3: Table S3.** Networks generated from gene expression data of restricted versus *ad libitum* fed bulls by IPA

| Network ID | | Top functions | Molecules in network | Score | Focus molecules |
| --- | --- | --- | --- | --- | --- |
| 1 | Hematological Disease, Lipid Metabolism, Molecular Transport | | *AAMDC, APP, C11orf71, C19orf12, C19orf43, CARS, CCDC25, CRYL1, DKKL1, DPY19L3, GALE, GIMAP4, KIAA0825, LLPH, MRPL20, MRPL52, MRPS18A, MTHFR, NDUFAF7, OSBPL11, PNPO, PROSER2, PRR16, RTN2, SGK2, SGMS1, SLC48A1, SPX, TCP11L2, TEX2, TRIM47, TSPAN13, WDR89, ZCCHC6, ZKSCAN1* | 49 | 35 |
| 2 | Protein Synthesis, RNA Post-Transcriptional Modification | | *FAU, POLG, PSPH, Ras, RASGRF2, Ribosomal 40s subunit, Rnr, RPS3, RPS5, RPS6, RPS7, RPS8, RPS9, RPS10, RPS11, RPS12, RPS13, RPS14, RPS15, RPS16, RPS18, RPS19, RPS20, RPS21, RPS23, RPS24, RPS25, RPS27, RPS28, RPS29, RPS15A, RPS27A, RPS3A, RPS4X, RPS4Y2* | 41 | 32 |
| 3 | Cardiovascular Disease, Developmental Disorder, Hematological Disease | | *AGL, BLMH, CUL4B, DCAF11, DDX3X, EEF1B2, GLTSCR2, HOOK1, MHC Class I (complex), phosphoinositide phospholipase C, PLCB1, PLCD4, PLCH1, PSTPIP1, Rab11, Rac, REV3L, RPL5, RPL6, RPL8, RPL10, RPL11, RPL13, RPL18, RPL23, RPL26, RPL28, RPL31, RPL10A, RPL7A, RPLP1, RPLP2, SAP130, TPT1, VPS41* | 39 | 31 |
| 4 | Hereditary Disorder, Neurological Disease, Carbohydrate Metabolism | | *20s proteasome, 26s Proteasome, ALAD, ASPSCR1, C/ebp, CD27, FAM188A, GCC2, IDE, IDH2, Ifnar, IKBKB, KIF20A, KIF21A, LOXL4, MAP1LC3, MAP1LC3A, MAT2A, MHC CLASS I (family), MYO1D, NHLRC1, NLRC5, NLRX1, NNAT, PGAP1, PHAX, PPP1R3B, PSMB3, PSMB8, PYGL, RAD23A, SLC19A1, TARS, TMEM160, VCPIP1* | 34 | 29 |
| 5 | Energy Production, Lipid Metabolism, Small Molecule Biochemistry | | *AADAC, ACOX1, APOA1, CARHSP1, CPT1A, CTSC, DDIT3, GALNT1, Growth hormone, HDL, Insulin, Ldh (complex), MAOB, NR0B2, OAT, PCYOX1, PPA1, PPARA, PTGR1, SAA, SAR1B, SARDH, Sec23, SETX, SGMS2, SIAH1, SLC37A4, SLC38A2, SPEF2, TF, TRIM7, UBD, Uox, UPP2, XDH* | 34 | 29 |
| 6 | Organismal Injury and Abnormalities, Renal and Urological Disease, Cell Cycle | | *14-3-3, AADAT, ADAMTSL4, Ant, ATXN1, BRCA1, Caspase 3/7, CMC1, CPNE3, DZIP3, FBXO42, FBXO44, FLRT2, GTF3C4, Holo RNA polymerase II, IGDCC4, INMT, KCNMA1, LZTS2, MBP, MRPL40, MTORC1, NATD1, NEBL, NELFE, NFYB, PAM16, RCL1, RPA, SLC23A1, SLC25A6, TCEA2, TCEB2, TLE4, TOMM6* | 34 | 29 |
| 7 | Energy Production, Lipid Metabolism, Small Molecule Biochemistry | | *3-hydroxyacyl-CoA dehydrogenase, ACOT8, ANKRD44, ANPEP, c-Src, CRAT, CRY1, DDAH1, EHHADH, GDPD5, Gli, HACL1, HADH, HADHB, HDC, Hedgehog, HSDL2, IDH1, IHH, IL17RC, KIAA1522, Mucin, NR1D1, NR1D2, NUDT19, P4HTM, Patched, Pde4, PEX5, SFRP1, SLC19A2, TDO2, Vegf, VEGFB, ZBTB10* | 30 | 27 |
| 8 | Cell Signaling, Lipid Metabolism, Small Molecule Biochemistry | | *Acox, ALDOB, ARHGEF19, B3GALT4, CABLES1, CEP83, CYP26A1, DAAM1, ENO3, ENO4, Enolase, EXTL3, GRASP, GSDMB, Hnf3, Igf, Igfbp, IGFBP1, IGFBP2, IGFBP6, Kallikrein, KCTD3, MAPRE3, ME1, MMP11, NUP210, RARG, RETSAT, RHPN2, RXRG, SLIRP, SRC, T3-TR-RXR, thyroid hormone receptor, VitaminD3-VDR-RXR* | 29 | 26 |
| 9 | Nervous System Development and Function, Organ Morphology, Organismal Development | | *AIM1, ANGPTL4, CBFA2T2, CDO1, Ctbp, DIO1, Ecm, ECM1, elastase, Fibrin, GGT1, Hdac, IFITM1, IFRD1, IGF1, LARGE, LHX2, MMP15, N-cor, NCOR1, NCOR2, PATZ1, PCSK5, PCSK6, RAB29, Rar, RARRES2, ROBO2, Rxr, SERPINA1, SPR, TCF, TCF21, THRSP, YARS* | 29 | 26 |
| 10 | Drug Metabolism, Molecular Transport, Lipid Metabolism | | *ABCB11, ABCC2, ABCC6, ABCD1, ATF5, ATP11B, ATP8B1, ATP9B, ATPase, BRCC3, Calmodulin, CARNS1, CDA, DAPK2, EEF2K, FETUB, FXR ligand-FXR-Retinoic acid-RXRα, HSPB1, IGSF5, Importin alpha, KDM4B, KIF1B, MAGI1, Mg2+-ATPase, MIB1, Nos, NOSIP, P glycoprotein, PFK, PGD, PPTC7, RAB40B, RASSF6, SWSAP1, Ubiquitin* | 29 | 26 |
| 11 | Post-Translational Modification, Small Molecule Biochemistry, Drug Metabolism | | *ACP5, AKR1D1, alcohol group acceptor phosphotransferase, ARSG, ASNS, Cyclin D, CYP39A1, DST, DYRK1A, DYRK1B, FAM20B, IL23, IL12 (family), JINK1/2, JUN/JUNB/JUND, MAP2K6, MAPK6, MAPK9, NOD2, PEPCK, PTX3, RORC, Secretase gamma, SLC30A10, SLC41A2, SLC7A2, Sult1a1, SULT1B1, SULT1E1, SULT2A1, thymidine kinase, TK2, TRAF3, TRAF3IP1, ZNF516* | 29 | 26 |
| 12 | Nucleic Acid Metabolism, Small Molecule Biochemistry, Hereditary Disorder | | *2-ketoglutarate dehydrogenase, AMIGO2, APC (complex), BEX2, BLVRB, Bvr, CCDC86, CD99, CITED4, CKAP2, DHTKD1, DLST, Fcgr2, FSD2, I kappa b kinase, IFT20, IFT27, IKBKAP, JAKMIP2, MAP3K, MRPL11, NFkB (complex), NIK, OGDH, ORMDL3, PDLIM1, Plk, PLK2, PLK3, PLP2, PPARα-RXRα, RUSC2, SLC2A5, TRMT112, UNC13D* | 27 | 25 |
| 13 | Cell-mediated Immune Response, Cellular Development, Cellular Function and Maintenance | | *APBB3, BEGAIN, BEND5, CD8, CD47, CD3-TCR, CD3E, CD3G, CD8B, CTNNBIP1, Fcgr3, FYN, growth factor receptor, HERC2, IGF2R, IL-2R, Integrinα, Integrinβ, LAT, LCK, Lfa-1, LRBA, NCKAP5, NEDD9, NFAT (complex), PLC gamma, PTPRCAP, RBM3, SLA2, SMPDL3B, TCF7, TFRC, XAF1, ZAP70, ZNF106* | 27 | 25 |
| 14 | Hematological Disease, Metabolic Disease, Developmental Disorder | | *ABCB10, AMT, BTG3, C1q, CCDC69, CPS1, DCPS, DIP2A, EXOSC4, FST, GADD45GIP1, GALNT10, GLDC, HBD, hemoglobin, IgG1, Igg3, IgG2a, IgG2b, INHBA, INHBC, INHBE, Inhibin, JMJD6, LGMN, MHC II, Mir122a,b, NAV3, SEMA4B, SH2D1A, SRC (family), TCIRG1, ZAK, ZC3H12A, ZNF593* | 27 | 25 |
| 15 | Auditory Disease, Cell Cycle, Cellular Assembly and Organization | | *ADAR, Alpha actin, APOL2, ASB9, ATG2A, CD14, chemokine, CKB, COMMD1, DNAJB1, DNAJB11, DNAJC12, DNAJC18, DNAJC22, HSP, Hsp70, Hsp90, Hsp22/Hsp40/Hsp90, HSPA6, Ifn gamma, Interferon alpha, LSM3, MLF2, MYH14, PLEC, RAD21L1, RPL22, SEC14L3, SELK, SLC5A1, SMC1A, snRNP, SNRPG, Tlr, ZMAT5* | 27 | 25 |
| 16 | Cell Signaling, Cell Cycle, Auditory Disease | | *AMFR, AMY2A, amylase, ARHGAP35, ARL4D, ATP synthase, CAMK2N1, CD3D, CKS2, DERL3, DLAT, E3 RING, ERK, Erm, FBXL14, Fgfr, Gap, HERP, HERPUD1, HEY1, IL17RB, INTERLEUKIN, ITPR2, MKNK2, PDK2, PRKD3, PTGFRN, RAP1GAP, RhoGap, RTKN, SMAD1/5, SNCAIP, STARD9, TAX1BP3, UBR1* | 25 | 24 |
| 17 | Antigen Presentation, Developmental Disorder, Hereditary Disorder | | *ABAT, ACVR1, AFF1, ALT, ARSA, CDK12, CFH, CLEC4G, COL1A2, DAB2IP, DAO, DECR1, ERO1B, ETS, FOSL2, GOT, GOT1, Hepatic Transaminase, HPGD, IFN alpha receptor, Mapk, mediator, PC, Pdi, PDXK, Plc beta, PRF1, RFX5, RFXANK, Smad2/3, Smad2/3-Smad4, SRA1, SRXN1, TEAD1, TEF* | 25 | 24 |
| 18 | Carbohydrate Metabolism, Cellular Function and Maintenance, Cell Death and Survival | | *AASS, CHPF, CIRBP, CLCN4, collagen, Collagen type I, Collagen type IV, cytochrome C, DPYD, DUB, Fgf, FGFR4, GHR, Hsp27, JAG1, KLF9, MDM1, Mmp, MMP23B, NME3, ONECUT1, OTUD6B, PARP, PDLIM2, Pkg, PRAP1, Tnf (family), TNFSF10, TNKS, TNKS2, TXN, UCHL1, USP2, USP46, ZC3HAV1* | 25 | 24 |
| 19 | Lipid Metabolism, Small Molecule Biochemistry, Cancer | | *ACAC, ACAP1, AGPAT9, Akt, AMPK, APOA4, BMP5, CPT1, creatine kinase, CREB3L3, CREBZF, CYP19, DDT, DGAT2, EIF4EBP1, EIF4EBP2, FNIP1, GPLD1, IRS, LPIN1, LPIN2, MTORC2, MVD, NDPK, PDZK1IP1, PHLPP1, PRKAA, RANBP1, RHEB, SDHA, SDHC, SIRT2, succinate dehydrogenase, TTC23, VLDL* | 23 | 23 |
| 20 | Lipid Metabolism, Small Molecule Biochemistry, Vitamin and Mineral Metabolism | | *ADH4, AKR1C3, AKR7A2, Aldose Reductase, APOC4, ARG2, arginase, CBR1, DCXR, DHCR24, ECE1, ELOVL6, Fascin, FDFT1, glutathione peroxidase, HMG CoA synthase, HMGCS2, INSIG1, LDL, LPCAT3, MAGED1, MPC1, NADH or NADPH:quinone oxidoreductase, NCOR-LXR-Oxysterol-RXR-9 cis RA, NFKBIZ, Nr1h, Pkc(s), SCAVENGER receptor CLASS A, SEC16B, SLC20A2, TTC32, UNC5, UNC5A, UNC5B, UNC5C* | 23 | 23 |
| 21 | Gene Expression, Protein Synthesis | | *60S ribosomal subunit, APC/APC2, CK1, Dishevelled, DKK, DKK3, Frizzled, Glycogen synthase, Gsk3, Histone H1, Importin beta, LRP6, RPL14, RPL15, RPL17, RPL21, RPL24, RPL27, RPL29, RPL32, RPL35, RPL36, RPL37, RPL38, RPL18A, RPL23A, RPL27A, RPL35A, RPL36A, RPL37A, SPATA13, TNPO1, Transportin, UBA52, Wnt* | 23 | 23 |
| 22 | Post-Translational Modification, Cellular Development, Cell Death and Survival | | *BCL2L2, Cg, CLSTN1, COX8A, DLC1, DUSP26, FOXO3, FSH, GADD45, Gamma tubulin, GOLGB1, IRS2, ITSN2, JAK, Lh, MIF4GD, NEK9, NMDA Receptor, Oas, PCED1A, PDE4DIP, PLC, PTEN, PTPase, PTPN7, PTPN9, PTPN21, SLC1A4, SLC4A4, Sod, STS, TMF1, TP53BP2, tyrosine kinase, YTHDF2* | 23 | 23 |
| 23 | Drug Metabolism, Small Molecule Biochemistry, Lipid Metabolism | | *ABCA5, APOA5, APOF, BRMS1L, Ces, CES2, CYP2C9, CYP2C19, CYP2E1, CYP2J2, CYP3A4, CYP4A, CYP4B1, FECH, Ferritin, glutathione transferase, GPC3, Gst alpha, GSTA4, GSTM1, GSTM3, GSTM4, HDL-cholesterol, HNF4α dimer, HPX, Jnk, LDL-cholesterol, MZB1, Ncoa-Nr1i2-Rxra, PAPSS2, PON1, PXR ligand-PXR-Retinoic acid-RXRα, SLC51B, unspecific monooxygenase, VLDL-cholesterol* | 22 | 22 |
| 24 | Cellular Assembly and Organization, Cellular Function and Maintenance, Tissue Development | | *Actin, Alpha Actinin, ATP2B2, ATP2C1, CALD1, CFL2, CLCN3, Cofilin, Collagen type V, F Actin, Fcer, Focal adhesion kinase, G-Actin, GAS8, HOMER2, Integrin alpha V beta 3, ITGA7, ITGAV, LIMA1, LIMS2, Pak, PARVA, PLD1, Pmca, PSD3, RASGRP2, Rho gdi, RHOC, SCIN, SORBS1, SWAP70, TESK1, TESK2, Tropomyosin, TXNRD1* | 22 | 22 |
| 25 | Molecular Transport, Small Molecule Biochemistry, Cancer | | ANP32B, APCDD1, BICC1, calpain, caspase, CCND3, Cdc2, CDCA7, Cdk, CDK11A, CDKN1B, CUX2, Cyclin A, Cyclin B, Cyclin E, cyclooxygenase, DHFR, E2f, GDA, HIST2H4A, Mek, MIF, PANX1, POLE, POMC, QKI, Raf, Rb, RBBP8, SLC16A6, Sos, TOB1, TOPBP1, TRMT13, UXT | 22 | 22 |
