# Supplementary material for: Effect of dietary restriction and subsequent re-alimentation on the transcriptional profile of hepatic tissue in cattle
Source: BMC Genomics. 2016 Mar 17;17:244. doi: 10.1186/s12864-016-2578-5 (PMC4794862; doi:10.1186/s12864-016-2578-5)
Supplement: Additional file 4: Table S4. — Networks generated from gene expression data of bulls undergoing compensatory growth versus ad libitum fed bulls by IPA. (DOCX 15 kb) [file 12864_2016_2578_MOESM4_ESM.docx]

**Additonal file 4: Table S4.** Networks generated from gene expression data of bulls undergoing compensatory growth versus *ad libitum* fed bulls by IPA

| Network ID | Top functions | Molecules in network | score | Focus molecules |
| --- | --- | --- | --- | --- |
| 1 | Cellular Compromise, Cellular Function and Maintenance, Neurological Disease | *Alp, calpain, COL1A1, COL1A2, Collagen type XVIII, Collagen(s), DDIT3, DERL2, DNAJB9, DNAJB11, ERK, ERK1/2, FADS1, FBLN1, FKBP9, GPBAR1, HERPUD1, HNF1B, HSP, Hsp70, HSPA5, Insulin, LDL, MAP2K1/2, MGP, P38 MAPK, PDGF BB, PI3K (complex), PI3K (family), Pro-inflammatory Cytokine, Proinsulin, Rxr, SREBF1, Tgf beta, Vegf* | 35 | 15 |
| 2 | Cell Death and Survival, Nucleic Acid Metabolism, Small Molecule Biochemistry | *ANKZF1, APP, ARPP19, ASGR1, B4GALT1, BCORL1, CAMKMT, CCDC134, CR1L,DDR2, HBD, HDAC4, HIF1A, HSP90AA1, IFNG, IgG1, KLHL32, LAMA4, MANF, MAPK1, MAST3, MIS12, MX2, NPPB, NT5E, PLA2G5, PRSS23, PTGIS, RFX5, RFXAP, SELK, SEMA4B, SGK2, TMEM45A, TNF* | 21 | 10 |
| 3 | Infectious Diseases, Hair and Skin Development and Function, Organismal Injury and Abnormalities | *AEBP1, Akt, B4GALT1, CD6, Ces1e, CR1L, DDX58, EZR, FSH, Histone h3, HSD3B2, IFNE, IgG, IGK, Interferon alpha, Jnk, KRT7, LGALS7/LGALS7B, Mapk, MX, MX2, NFkB (complex), NUS1, PARVG, Pde4, Pka, Pkc(s), PODXL, PPP1R1A, PPP1R1B, Ras, SIGLEC10, SLC12A7, TCR, TSC22D3* | 16 | 8 |
| 4 | Cell Morphology, Cellular Growth and Proliferation, Post-Translational Modification | *CBX4, RB1, RNF111, SKIL, THAP5, UBC, UBE2I* | 4 | 2 |
| 5 | Cell Cycle, Cell-To-Cell Signaling and Interaction, Nervous System Development and Function | *ARHGAP30, ITSN1, SMARCA4* | 2 | 1 |
| 6 | Gene Expression, Nervous System Development and Function, Carbohydrate Metabolism | *Holo RNA polymerase II, PPARD, RPH3AL, SFTPA1, TCEA3, THRB* | 2 | 1 |
| 7 | Cell Cycle, DNA Replication, Recombination, and Repair, Cellular Assembly and Organization | *CHERP, DHX16, MEPCE, PRPF4, PRPF8, snRNP, SNRNP27, SRPK2, STXBP5L, USP39* | 2 | 1 |
